# Students’ motives for restricting academic freedom: Viewpoint discrimination and prosocial concerns

**DOI:** 10.1073/pnas.2503804122

**Published:** 2025-11-20

**Authors:** Claudia Diehl, Matthias Revers, Richard Traunmüller, Nils B. Weidmann, Alexander Wuttke

**Affiliations:** ^a^Department of History, Sociology, Educational Science and Sports, University of Konstanz, Konstanz 78457, Germany; ^b^Institute of Journalism and Communication Studies, University of Hamburg, Hamburg 20146, Germany; ^c^School of Media and Communication, University of Leeds, Leeds LS2 9JT, United Kingdom; ^d^School of Social Sciences, University of Mannheim, Mannheim 68159, Germany; ^e^Department of Politics and Public Administration, University of Konstanz, Konstanz 78457, Germany; ^f^Geschwister-Scholl-Institute of Political Science, Ludwig Maximilian University Munich, Munich 80539, Germany

**Keywords:** academic freedom, adversarial collaboration, cancel culture, survey experiment, higher education

## Abstract

We advance the understanding of so-called “cancel culture” at the university by presenting the results of three survey experiments among university students. Designed in an “adversarial collaboration” among researchers with competing perspectives, these experiments disentangle whether students’ preferences for curtailing academic freedom are based on viewpoint discrimination, professional academic standards, or prosocial concerns. Our findings show that a substantive share of university students support viewpoint-based restrictions on academic discourse. While they also apply academic and prosocial criteria, they apply them more strongly to conservative viewpoints. The results further show that conservative statements are perceived as causing more social harm. However, prosocial concerns do not fully explain the higher demand for ideological viewpoint discrimination. These results are important because they can inform the debate about universities as ideological spaces—a view often invoked in recent government-led attacks on academic freedom.

## Significance

Academic freedom is under threat across the globe and is of vital concern to both researchers and society. We show that while limits on academic freedom are partly motivated by prosocial concerns, political viewpoint discrimination is another important driver of restrictions placed on speakers, teachers, and books on university campus. These findings are of great significance because they inform the debate about universities as ideologically biased environments—a view often invoked in recent government-led attacks on academic institutions.

Free expression at the university has long been at the center of a fierce debate. Controversy revolves around who should have the right to speak on university campuses, who should be allowed to teach, which books should be banned, and how to deal with disruptive student protests. While some observers have diagnosed an increasingly restrictive atmosphere and an alleged cancel-culture on college campuses (1, 2), others have welcomed a new sensitivity which turns universities into more inclusive spaces (3). Most recently, portraying universities as ideologically biased has served to justify government-led attacks on academic institutions.

We advance this debate by presenting the results of three preregistered experiments among university students. Building on seminal work in political psychology (4, 5), our experimental study expands on recent student surveys which generally show low support for free speech in the US university context (6, 7) and beyond (8). While these studies contribute significantly to our understanding of university students’ preferences, they say little about students’ underlying motives for restricting academic freedom (but see ref. 9). Students’ motives are important, as many cancellation attempts have long been driven by their demands (10). Understanding these motives is also essential for making normative evaluations and devising appropriate responses to such events.

When debating the rationale for protecting or restricting free expression at the university, it is widely agreed that academic freedom has the function to protect research and teaching from ideological, political, or religious interference (11–13). Academic discourse therefore should not be restricted based on *viewpoint discrimination*. However, academic freedom is not the same as free speech in general (14) and therefore stricter limits on the former are warranted. If academic institutions are to fulfill their societal role of discovering and disseminating knowledge, free expression in the academic context must be restricted by shared *academic standards* and practices of quality control (15). A further, and more contested, position holds that restrictions on academic freedom may also stem from *prosocial concerns*—that is, the worry that research will be used to support harmful policies or directly harms vulnerable groups (10, 16).

To establish whether viewpoint discrimination, academic standards, or prosocial concerns drive students’ preferences for restricting academic freedom, we experimentally manipulate statements expressed in hypothetical talks in a university context. To overcome the limitations of previous studies of viewpoint discrimination, we carefully select ideologically balanced viewpoints that are currently considered controversial (17) without being unequivocally hateful (7). For each statement, we randomize two *ideologically* coded versions—one progressive, one conservative (18)—, vary the extent to which it conforms to *academic standards*, and manipulate whether it raises *prosocial concerns*.

Overall, our findings suggest that a substantive share of university students support viewpoint-based restrictions on academic discourse. Although they also apply professional-academic and prosocial criteria, they do so more strongly for conservative viewpoints. While conservative statements are often perceived as more socially harmful, this perception alone does not fully account for the stronger demand to restrict conservative speakers, teachers, or books. Instead, this demand appears to be equally driven by a rejection of certain ideological positions, i.e., viewpoint discrimination.

## Adversarial Collaboration

Given that academic freedom and its limits are the subject of polarized debate, we strengthen the credibility of our research with the emerging open science practice of adversarial collaboration (19). The objective of this research practice is to reach a consensus on research design and inference criteria among competing scholars who disagree in their outlook and expectations. Our project aims to extend and improve earlier empirical work that has sparked controversy (8), leading to a collaboration between its original authors (MR and RT—the proponents in the current project), scholars who disagree with the original conclusions and propose revisions (CD and NW—the critics), and an impartial referee (AW).

We preregistered four hypotheses concerning students’ motivations for supporting cancellation and for which the proponents and critics held competing prior expectations (Table 1). The proponents expect that students’ support for cancellation is primarily driven by discrimination against conservative viewpoints. In contrast, the critics expect that students are more nuanced in their judgments and primarily motivated by a commitment to professional academic standards and prosocial concerns about the potential political and social harm of certain views, particularly to vulnerable groups on campus. In addition, the proponents preregistered a—necessarily somewhat arbitrary—benchmark of 20% as a problematic level of support for restrictions on academic freedom (see the *SI Appendix* for more details on the adversarial collaboration).

**Table 1.** Preregistered hypotheses and prior expectations of the adversarial collaboration

Motivation for restricting academic freedom	Proponents’ expectations: Students’ support for restrictions…	Critics’ expectations: Students’ support for restrictions…
Viewpoint discrimination (H1)	… is higher for conservative viewpoints than for progressive viewpoints.	… does not differ between conservative viewpoints and progressive viewpoints.
Academic standards (H2)	… does not differ between mere opinions and research results.	… is higher for mere opinions.
Prosocial concerns (H3 and H4)	… does not differ between statements with or without policy recommendations. … does not differ between statements that are criticized by groups on campus and those that are not.	… is higher for statements with policy recommendations.… is higher for statements that are criticized by groups on campus.

The table summarizes the theoretical expectations of the team of proponents and the team of critics.

## Study 1: Students’ Motives for Restricting Academic Freedom

We conducted a survey experiment to test the four hypotheses outlined in Table 1. University students were asked to rate vignettes containing fictional descriptions of speaking events on campus. Each vignette varied along four experimental dimensions: a) the ideological content of speech (progressive vs. conservative), b) the academic standards (research by professor vs. opinion by a journalist), and two types of prosocial concerns—whether c) a policy recommendation derived from the talk, and d) whether the statement faced criticism from groups on campus.

This design allows us to disentangle the extent to which viewpoint discrimination influences respondents’ support for various university actions in response to the event: Whether the university should a) cancel the talk, b) rescind a teaching offer to the speaker, c) remove the speaker’s book from the library, and d) permit protests around the event. Details of the research design, exact vignette wording, preanalysis plan, and ethics review are provided in *Materials and Methods* section and in *SI Appendix*.

**Fig. 1:**
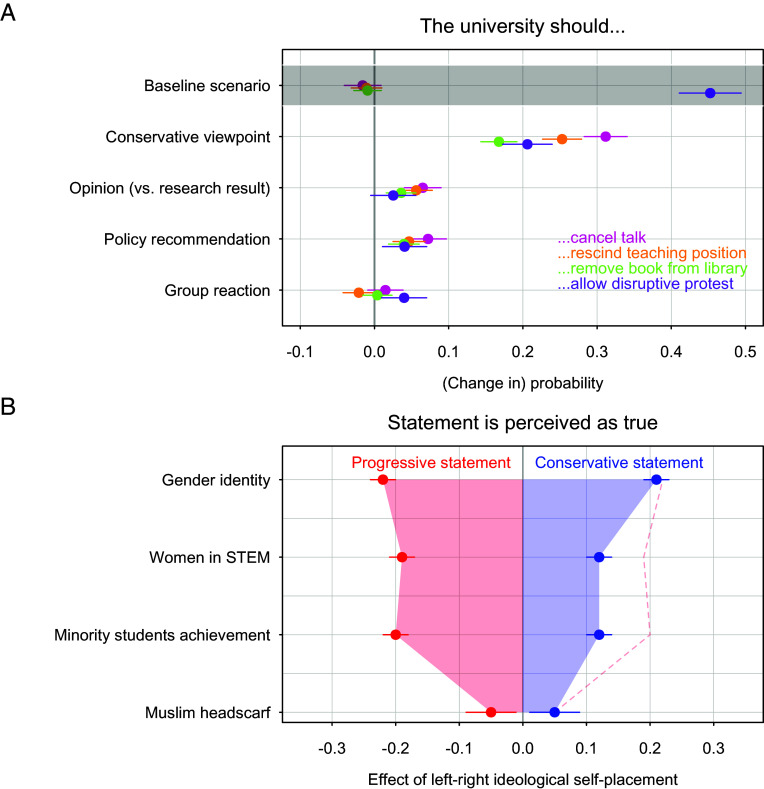
Main results of the vignette experiment. (*A*) Estimated effects of conservative (vs. progressive) viewpoint, journalist’s opinion (vs. professor’s research), inclusion of a policy recommendation, and criticism from groups on campus on support for university action against the speaker or event. Coefficients and 95% CI are from a linear probability model with clustered SE and vignette topic fixed effects. The baseline scenario (intercept) corresponds to progressive research without a policy recommendation and without criticism from groups on campus. Detailed results are provided in *SI Appendix*, Table S9. (*B*) Testing the ideological balance of vignette statements. The figure shows coefficients of ideological self-placement and 95% CI from a linear probability model predicting the perception that statements are true. A statement is considered balanced if the progressive version is perceived as true by conservative students to roughly the same extent that the conservative version is perceived as true by progressive students. Detailed results are provided in *SI Appendix*, Table S10.

Fig. 1*A* shows the main results of our vignette experiment. We find little evidence of speech restriction in the baseline scenario—a professor presenting research results that align with progressive views without policy recommendation and without any criticism from groups on campus. Deviations from the baseline scenario significantly increase students’ support for restricting expression on campus.

Students are particularly less tolerant when the content of the talk aligns with *conservative viewpoints*. Compared to the baseline (averaged across the four topic areas), support for canceling talks increases by 31 percentage points, support for rescinding teaching positions by 25 percentage points, and support for removing books by 17 percentage points when the viewpoint is conservative. The share of students who would allow protests increases by 21 percentage points.

Professional academic standards also matter to students, who clearly distinguish between personal opinion and academic research. When invited speakers are journalists presenting opinions rather than professors presenting research findings, students are more supportive of canceling talks (+7 percentage points), rescinding teaching positions (+6 percentage points), and removing books from the library (+4 percentage points, averaged across the four topic areas). There is no corresponding increase in support for protests. The effects of *prosocial concerns* yield mixed results. On one hand, students are more likely to support canceling talks (+7 percentage points), rescinding teaching positions (+5 percentage points), removing books (+4 percentage points), and allowing protests (+4 percentage points) when the speaker makes explicit policy recommendations. On the other hand, while students are more supportive of protests when groups on campus criticize the talk as offensive (+4 percentage points), the other three indicators of restricting academic freedom remain unaffected.

Our results are not driven by ideological imbalance in the construction of our vignettes. Fig. 1*B* shows that the conservative and progressive versions of the items are not perfectly balanced. We repeat the main analysis with the two balanced vignettes (gender identity and Muslim headscarf, see *SI Appendix*, Table S11) and obtained similar results. Additional robustness checks confirm the stability of our findings when applying survey weights (preregistered; *SI Appendix*, Table S12), including an index of respondents’ attentiveness (preregistered; *SI Appendix*, Table S13), and estimating logistic regression models to account for the binary nature of the outcome variables (not preregistered, *SI Appendix*, Table S14).

We also address the possibility that our main results are driven by an ideological imbalance in our sample. As expected in a university student population, the sample includes a higher proportion of progressive students: Based on self-placement on a 1 to 10 left–right scale, 70% identify as left-leaning students (self-placement of 5 or below) and 30% as right-leaning (6 or above). This imbalance may contribute to the stronger rejection of conservative statements. Is this a limitation? One could argue that this left-leaning tilt of our sample reflects the actual political composition of university campuses. Nonetheless, it remains important to assess whether the observed discrimination against conservative viewpoints is solely a function of this distribution.

To address this concern, we conduct two additional analyses: First, we control for ideological self-placement and find similar results (*SI Appendix*, Table S15). Second, we reweight the data to simulate a 50:50 distribution of left-leaning and right-leaning students; the estimated vignette effects remain consistent with the main results (*SI Appendix*, Table S16). We further confirm the robustness of our findings by controlling for other potentially unbalanced sample characteristics associated with more left-leaning attitudes among respondents (*SI Appendix*, Tables S17 and S18). Taken together, these results suggest that the observed willingness to cancel is not simply a product of universities’ political leanings but rather reflects a lower tolerance for opposing views among progressive students compared to their conservative peers.

Further exploratory analyses (not preregistered) reveal that the strong effect of *conservative viewpoints* interacts with other experimental dimensions. The results show that both the *academic standards* motive—rejecting statements framed as journalistic opinion (*SI Appendix*, Table S19)—and the *prosocial concerns* over policy recommendations (*SI Appendix*, Table S20) are applied only to conservative, but not to progressive, viewpoints. No such difference is observed for the information on criticism from groups on campus, where neither the main effects nor the interaction effects are statistically significant (*SI Appendix*, Table S21).

**Fig. 2.**
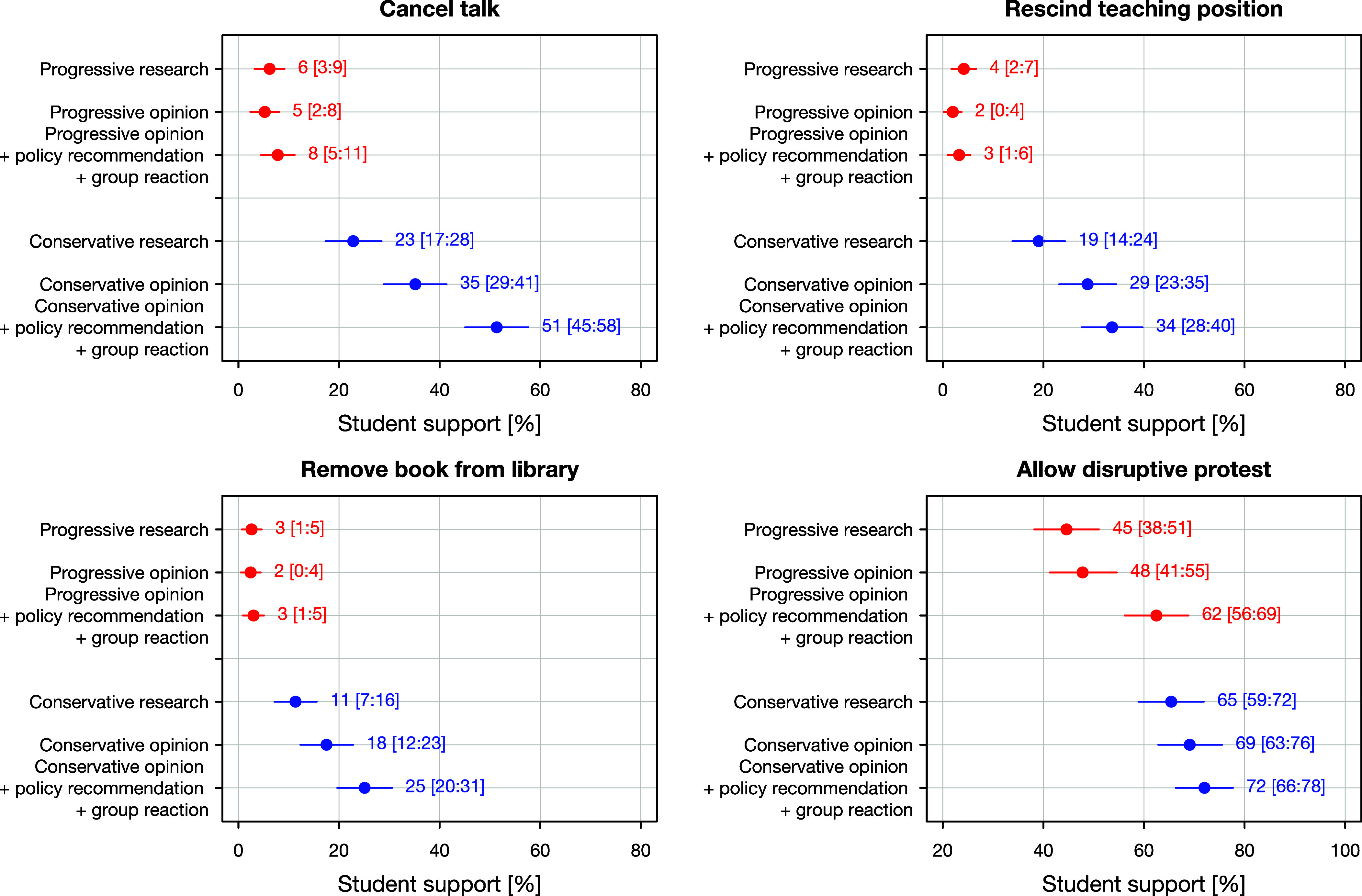
Predicted probabilities of support for canceling talks, rescinding teaching positions, removing books, and allowing protests. Marginal effects and simulated 95% CI are based on a linear probability model with clustered SE and vignette-topic fixed effects. Detailed results are provided in *SI Appendix*, Table S22.

To illustrate the substantive magnitude of students’ support for restricting academic freedom, Fig. 2 presents predicted probabilities for several key comparisons, based on models that include all interaction terms (*SI Appendix*, Table S22). Only a small percentage of students support restricting research results that align with progressive views (canceling talks: 6%; rescinding teaching positions: 4%; removing books: 3%), although many tolerate protests (45%). These proportions remain virtually unchanged even when comparing “progressive” research to progressive opinion, or to progressive opinion that includes explicit policy recommendations and draws criticism from groups on campus. One exception is protests, which about 62% of students support in the latter scenario.

By contrast, research results that align with conservative viewpoints are substantially less tolerated. Nearly one in four students (23%) supports canceling such a research presentation—even when it includes no explicit policy recommendation, and elicits no complaints from groups on campus. Then, 19% support revoking teaching positions, 11% support removing books, and 65% would allow protests. Thus, two of the four estimates (canceling talks and allowing protests) exceed the preregistered benchmark of 20% identified by the group of proponents as indicating a problematic level of support for restrictions at universities. A third estimate—revoking teaching positions—falls just below the threshold. Note that the critical benchmark is also exceeded for the progressive statement regarding the outcome of “allowing disruptive protest.” The latter is a type of restriction that generally receives high support from students. Support for restricting conservative viewpoints increases further when the content shifts from research to opinion, especially when accompanied by a policy recommendation and criticism from groups on campus. In this scenario, a majority of students (51%) support canceling the conservative talk.

Arguably, the most revealing comparison in Fig. 2 is between research findings that align with conservative viewpoints—i.e., research results without policy recommendations or criticism from groups on campus—and progressive opinions not based on research that include explicit policy claims and draw criticism from groups on campus. Across all indicators, students are more willing to restrict the former than the latter—often by a wide margin.

## Study 2: Ideological Viewpoints and Perceptions of Social Harm

Dismissing social harm as a relevant motive may be premature due to the design of the experimental variation in Study 1. In contrast to the policy implications, which explicitly distinguish between conservative and progressive proposals, the manipulation involving criticism from groups on campus makes no such distinction and does not specify the potential harm the statement could cause to these groups. This limitation may explain why this manipulation did not yield significant effects in Study 1.

More importantly, the social harm to vulnerable groups associated with conservative statements may be more severe and more salient in the public debate than the harm associated with progressive statements.^*^ For example, the (conservative) statement that gender is biologically determined may ultimately foster discrimination against trans people, whereas the (progressive) statement that gender is determined by one’s identity may appear to pose no comparable negative consequences for vulnerable groups. As a result, respondents may reject the former not due to viewpoint discrimination but because of prosocial concerns (i.e., anticipated harmful social consequences).

Therefore, our aim in Study 2 is to present social harm as a separate experimental treatment in both a conservative and a progressive version. We include only the two ideologically balanced vignettes from Study 1 (gender identity and Muslim headscarf), along with a new vignette on a highly timely and controversial topic (Israel vs. Palestine). For these vignettes, we pretested whether the described harm was perceived as similarly severe. This was largely—but not perfectly—the case for two of the three vignettes (see *Materials and Methods* section, *SI Appendix*, Fig. S1 and Table S28).

If the rejection of conservative items in Study 1 was primarily driven by concerns about social harm, then we would expect that providing information about a statement’s harmful consequences increases students’ willingness to impose restrictions on academic freedom *more* for progressive statements than for conservative ones—since the latter are already perceived as harmful. As a result, the difference in support for restricting academic freedom between conservative and progressive statements found in Study 1 should be reduced. In Study 2, both proponents and critics maintain their original hypotheses that support for restrictions is driven by ideology (proponents) vs. prosocial concerns (critics), and that these explanations remain valid even when specific information about potential social harm is added to the vignettes.

**Fig. 3.**
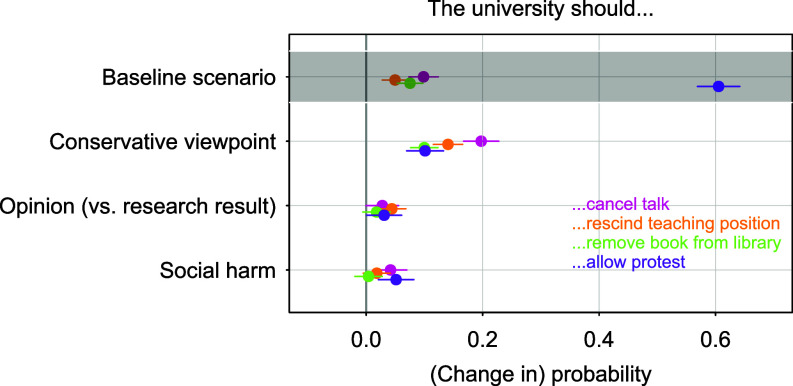
Main results of the second vignette experiment. Estimated effects of journalist opinion (vs. professor research), conservative (vs. progressive) viewpoint, and the presence of potential social harm associated with the speaker’s viewpoint. Coefficients and 95% CI are from a linear probability model with clustered SE and vignette topic fixed effects. The baseline scenario (first line) corresponds to progressive research results without an explicit mention of harm. Detailed results are provided in *SI Appendix*, Table S30.

Findings presented in Fig. 3 are overall consistent with those from Study 1. Averaging across the three topics—gender identity, Muslim headscarf, and Israel vs. Palestine—only a small share of students supports restrictions on freedom of speech in the baseline scenario in which a professor presents research findings that align with progressive views and no potential social harm is made explicit.

As before, viewpoint discrimination remains the strongest driver of students’ willingness to restrict academic freedom, although the effects are somewhat smaller than in Study 1. Compared to progressive viewpoints, conservative stances significantly increase support for canceling talks (+20 percentage points), rescinding teaching positions (+14 percentage points), removing books from the university library (+10 percentage points), and allowing protests (+10 percentage points). Once again, the distinction between opinion and research has little effect on students’ support for restrictions on free expression. Taken together, these findings indicate a successful replication of the main patterns from Study 1 on a different sample of students.

Regarding social harm, the results show that *prosocial concerns* about the potential harm of a speaker’s viewpoints have little influence on students’ willingness to curtail academic freedom. This finding from Study 1 holds even when explicit and specific information about potential social harm is provided for both progressive and conservative statements. Such information increases support for canceling a talk by only 4 percentage points and for allowing protests by 5 percentage points, with no significant effects for rescinding teaching positions or removing books. We again test whether our results could be driven by the uneven distribution of political preferences in our sample, which included more left- than right-leaning students. As in Study 1, we first estimate models that control for ideological self-placement and find similar results (*SI Appendix*, Table S32). We also apply weights to simulate a fully ideologically balanced sample of students; results in *SI Appendix*, Table S33 confirm that our results remain robust. In sum, viewpoint discrimination outweighs both academic standards and prosocial concerns—by an order of magnitude.

We now turn to the key question of Study 2: Do students reject conservative statements more often because they automatically assume these to be more socially harmful, while overlooking the potential harm of progressive statements? If this were the case, providing explicit and specific information about the social harm of both conservative and progressive statements should increase support for restrictions primarily in the latter case. However, when we interact the conservative treatment with the social harm treatment (*SI Appendix*, Table S31), we find no evidence that the effect of social harm depends on the ideological direction of the statement.

**Fig. 4.**
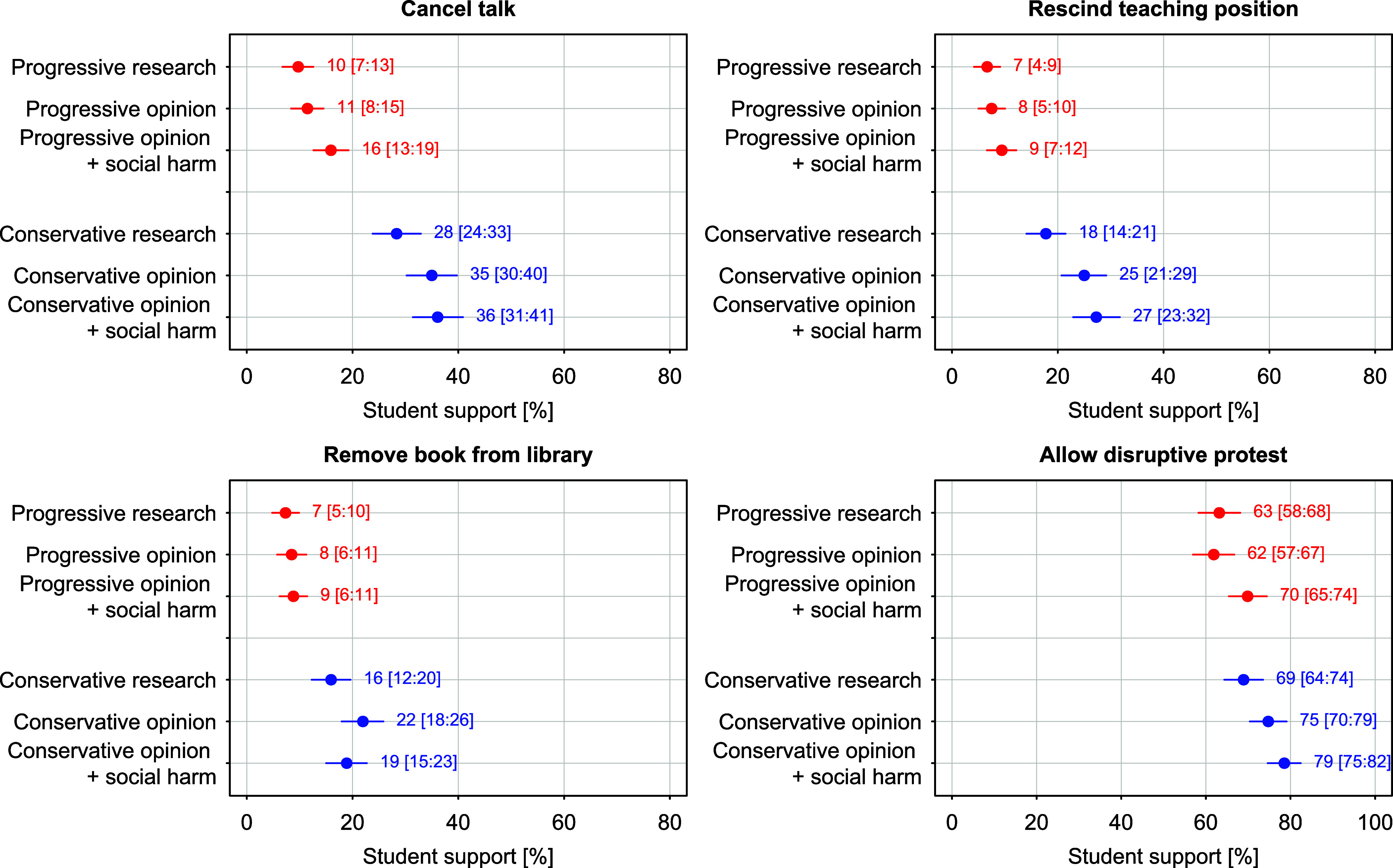
Predicted probabilities for support of canceling talks, rescinding teaching positions, removing books, and allowing protests in Study 2. Marginal effects and simulated 95% CI are based on a linear probability model with clustered SE and vignette-topic fixed effects. Detailed results are provided in *SI Appendix*, Table S34.

Fig. 4 presents predicted probabilities for several key comparisons in Study 2. These findings confirm the main conclusion from Study 1, although the difference in demand for restrictions on progressive and conservative speakers is less pronounced. Again, academic standards are not applied uniformly. The most instructive comparison is between progressive statements framed as opinion, explicitly referencing potential social harm, and research findings that align with conservative views. Conservative research is nearly twice as likely to be subject of cancellation as progressive opinion that includes a social harm cue. Whereas 16% of students would cancel a talk by a journalist expressing progressive and potentially harmful views, 28% would cancel a talk by a professor presenting conservative research findings. Similarly, about 9% support rescinding the teaching position and removing the book of a journalist with progressive and potentially harmful views, while 18% would revoke the teaching position and 16% would remove the book when the speaker is a professor presenting research findings that align with conservative viewpoints. No significant differences are observed for support of protests. Once again, two of the four estimates for the conservative statement (canceling talks and allowing protests) and one estimate for the progressive statement (allowing protests) exceed the critical benchmark of 20%, which the proponents defined as indicating a problematic level of restriction. Note that support for revoking the conservative speaker’s teaching position falls just below the threshold.

## Study 3: Do Prosocial Concerns Drive Viewpoint Discrimination?

Studies 1 and 2 both conclude that viewpoint discrimination is the main reason why students are willing to restrict academic freedom. However, neither study includes an assessment of the respondents’ *own* perceived harmfulness of the statements provided in the hypothetical scenarios. Consequently, we cannot rule out that what looks like viewpoint discrimination in our experiments is still driven by respondent’s personal prosocial concerns. To address this limitation, we replicate the vignette experiment from Study 2 using a new sample of university students and demonstrate that the general patterns remain consistent (*SI Appendix*, Figs. S2 and S3). But in addition, we now also ask respondents to rate, after each vignette, i) the harmfulness as well as ii) the political position of the presented statements. This helps us to identify the mechanism that drives the greater willingness to cancel conservative viewpoints.

**Fig. 5.**
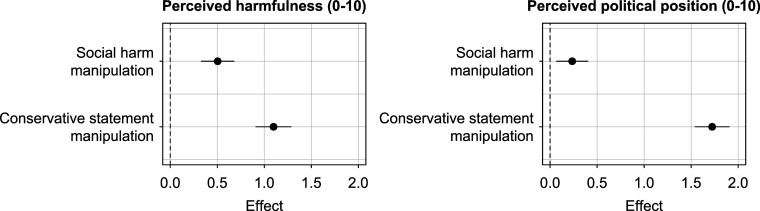
Manipulation check. Effects of experimental manipulations on perceived harmfulness ratings (*Left*) and perceived political position (*Right*). Detailed results are provided in *SI Appendix*, Table S38.

Based on these new data, we first conduct a manipulation check by regressing respondents’ own ratings on the “conservative statement” and “social harm” experimental manipulations. Ideally, we would observe that the conservative statement manipulation has a strong and significant effect on the political position rating but no effect on the harmfulness rating, and that the social harm manipulation has a strong and significant effect on the harmfulness rating but no effect on the political position rating. The results of the manipulation check in Fig. 5 confirm that the experimental conditions largely affect the intended constructs. As expected, the harm manipulation increases perceived harmfulness (by about 0.5 scale points) and the conservative statement manipulation shifts perceived political position to the right (by about 1.7 scale points). But the manipulation check also reveals notable spillovers: Making harm explicit also pushes political position ratings to the right and the conservative statement manipulation also increases perceived harmfulness by a whole scale point. In short, student respondents, on average, associate conservative views with more social harm.

In a second step, we therefore conduct a causal mediation analysis to isolate the average direct effect (ADE) of the conservative statement manipulation from the average indirect or causal mediation effect (ACME) which runs via perceived harmfulness (an analysis that is not without issues, see *Materials and Methods* for the required assumptions and their potential violation). The ADE is the effect of the conservative statement without any contribution of the harmfulness rating and thus provides a “clean” estimate of viewpoint discrimination. We will compare this quantity to the ACME which attributes the effect of conservative statements to their perceived harmfulness and thus provides evidence for prosocial concerns. If the proponents are correct and the willingness to restrict academic freedom is based on viewpoint discrimination, we should observe a significant ADE and no significant ACME. If the critics are correct, we should see the opposite. A weaker version of resolving these competing hypotheses would be an effect size comparison: If the proponents are correct, the ADE should be stronger than the ACME (and vice versa if the critics are correct).

**Fig. 6.**
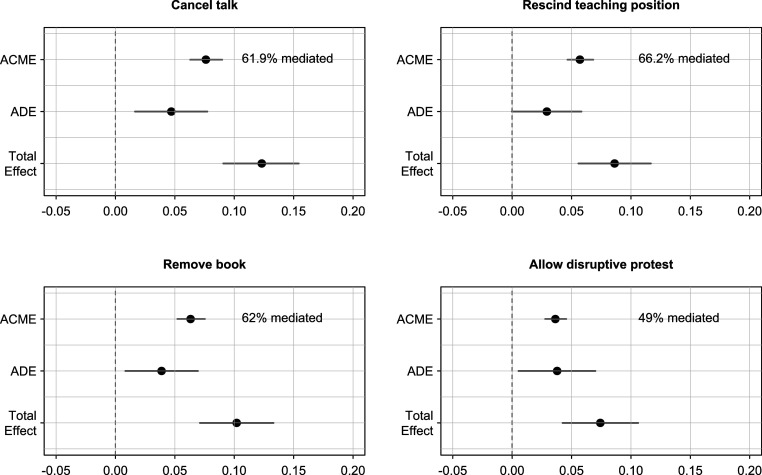
Causal mediation analysis. ACME and ADE of conservative statements on the willingness to cancel. Quantities are derived from the results in *SI Appendix*, Tables S42 and S43. 95% CI are based on 10,000 quasi-Bayesian simulations. Numerical results for the ACMEs, ADEs, and Total Effects are provided in *SI Appendix*, Table S44.

Fig. 6 presents the results of the causal mediation analysis and shows how the total effect of conservative statements on the support for restrictions can be decomposed. We find that, depending on the outcome, between 49% and 66% of this total effect is mediated by perceived harmfulness. The ACMEs are significant throughout and suggest that prosocial concerns are an important mediator of the effect of conservative statements on the willingness to restrict academic freedom. Further analyses show that this indirect effect is due to the higher levels of perceived harmfulness for conservative viewpoints, and not to a greater effect of their perceived harmfulness on canceling (*SI Appendix*, Table S43). Yet, we also find significant ADEs, which demonstrates that conservative statements are still more likely to be cancelled—even when accounting for their higher perceived social harm. Thus, the effect of conservative statements established in Studies 1 and 2 cannot be reduced to personal prosocial concerns, but also reflects ideological viewpoint discrimination to a considerable degree. In terms of effect sizes, the ACMEs and ADEs (and therefore prosocial concerns and viewpoint discrimination) do not differ significantly. Therefore, the evidence supports both the proponents and the critics.

## Conclusion

Our adversarial collaboration reveals that a substantial number of German university students support restrictions on academic debate on campus, including the cancellation of talks, the revocation of teaching positions, and the removal of books. Many also endorse disruptive protests against controversial speakers. Our experimental evidence shows that viewpoint discrimination is a key motivator behind these student demands. While the desire to restrict academic freedom also reflects an interest in upholding professional academic standards and concerns about the political and social implications of academic research, these principles are applied unevenly and predominantly to conservative viewpoints.

Our experimental designs allow us to rule out several alternative explanations for the greater willingness to restrict conservative over progressive viewpoints at the university. First, this pattern cannot be attributed to an ideological imbalance in the viewpoints presented in the vignette descriptions. Second, our results are not driven by the ideologically skewed composition of either the student sample or the broader student population. Third, viewpoint discrimination can only partly be explained by concerns about the social harm associated with conservative positions. The team of critics hypothesized that conservative statements are more likely to be canceled because they are perceived as causing greater harm to vulnerable groups. While the evidence partly supports this expectation, the findings also show that a statement’s conservative orientation increases the willingness to restrict academic freedom, independently of its perceived harm to vulnerable groups (the proponents’ expectation). Additionally, we wish to note that the degree of harm measured in our study constitutes a subjective perception rather than an objective assessment and is therefore itself not free from ideological bias.

In sum, our findings complement and qualify recent studies on prosocial academic censorship (9, 10) by demonstrating that restrictions of academic freedom are also, and to a considerable degree, driven by ideological viewpoint discrimination. This aligns with scholarship that identifies ideological conflict as an important mechanism underlying intolerance (20–22) and finds that conservative viewpoints are often deemed harmful (23). To be sure, German universities benefit from a relatively robust, state-funded, and state-sanctioned institutional framework that does not easily yield to students’ demands. However, whether this is sufficient to safeguard academic freedom from ideological attacks across the political spectrum remains uncertain. Indeed, it may pose an even greater risk when such attacks are initiated by governments themselves.

## Materials and Methods

### Study 1.

We obtained ethics approval and informed consent (EK 28/2023, University of Mannheim) and preregistered our analyses (see details in *SI Appendix*). The experimental setup presents each respondent with four fictional examples of planned university talks. We selected four controversial topics: gender identity, women in STEM, minority student disadvantage, and the Muslim headscarf. Unlike earlier approaches (24), we experimentally manipulated several characteristics of the prospective talk, resulting in a 2^4^ mixed design. Table 2 illustrates the design with an example vignette. Further details on vignette construction and exact wording are provided in *SI Appendix*, Table S1, along with a power analysis.

**Table 2.** Example vignette with the four different experimental dimensions

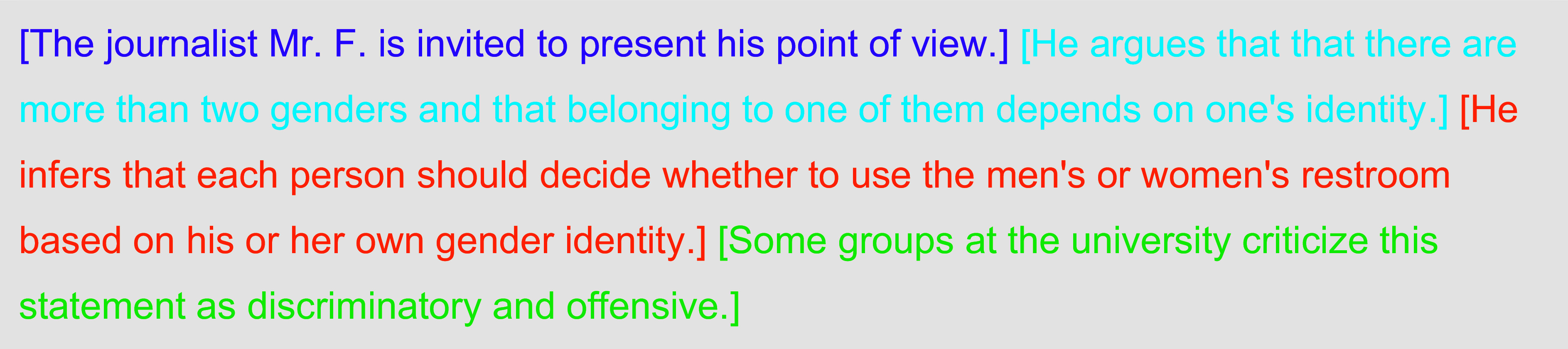

Academic standards (purple), content (blue), policy implication (orange), and criticism from groups on campus (green).

The experiment was embedded in a representative survey of *N* = 1,003 university students in Germany. While the debate about free expression is less pervasive in Germany than in the United States, it has gained prominence in recent years. Unlike the United States, with its strong First Amendment tradition, Germany maintains a broad consensus in favor of banning certain forms of speech, including expressions that are antisemitic or that trivialize Nazism (25, 26). Data collection took place between May and July 2023. Compared to registry data on the German student population, the sample closely reflects the population in terms of disciplinary composition, gender, and geographic distribution (*SI Appendix*, Table S2). In the *SI Appendix*, we report balance checks (*SI Appendix*, Tables S3–S6) as well as attention and manipulation checks (*SI Appendix*, Tables S7 and S8).

### Study 2.

We obtained informed consent and ethics approval (EK 16/2025, University of Mannheim) and preregistered our analyses (see details in *SI Appendix*). In Study 2, we retained the two ideologically most balanced statements from Study 1 (Islamic headscarf, gender identity) and introduced a new one (Israel vs. Palestine). Since our focus was to isolate the effects of perceived social harm, we omitted the policy recommendation manipulation used in Study 1. Instead, our revised prosocial concern treatment provides specific information about the potential social harm that conservative and progressive statements could entail for vulnerable groups (*SI Appendix*, Table S23).

We pretested whether the statements were balanced in terms of political orientation in a separate sample of *N* = 620 respondents (see the *SI Appendix* for details) and found that progressive and conservative statements were indeed perceived as such (*SI Appendix*, Table S27). Importantly, the statements were also rated for perceived social harmfulness on a 0 to 10 scale (*SI Appendix*, Fig. S1). Although average harmfulness ratings were slightly higher for conservative than for progressive statements (by 1.0 to 1.9 scale points), these ratings reflect subjective perceptions and are biased by the ideological composition of the sample, which was skewed in favor of left-leaning respondents (75:25 ratio). When rebalancing the sample to a 50:50 left–right distribution, the gap in the perceived harm decreased to approximately half a scale point (*SI Appendix*, Fig. S1, *Right*). We considered statements to be equally harmful when the absolute magnitude of the regression coefficients for individual ideology is the same for both conservative and progressive versions. This condition holds for the gender identity and Israel/Palestine vignettes, but not for the Muslim headscarf vignette (*SI Appendix*, Table S28). We surveyed *N* = 1,117 German students for Study 2. Further details on the study design are provided in *SI Appendix*.

### Study 3.

We obtained informed consent and ethics approval (Supplement 1 to EK 16/2025, University of Mannheim) and preregistered our analyses (see details in *SI Appendix*). Study 3 is a replication of the revised vignette experiment of Study 2. Next to using a fresh student sample, this replication makes two important additions. First, it provides a more direct manipulation check by adding two rating questions about each vignette’s perceived harmfulness and political position, i.e., this replication elicits the ratings directly from the individual respondents of the vignette experiment. Second, the replication presents a causal mediation analysis to compare the ADE of the ideology manipulation to its (potential) indirect or ACME via harm perceptions. To derive these two quantities, we rely on the mediation equations in *SI Appendix*, Table S43 and on the outcome equations in *SI Appendix*, Table S42. Causal mediation analysis rests on a set of assumptions, notably no unobserved confounding of all causal paths or “sequential ignorability” (27). This assumption is met for the paths running from conservative statement to the four outcomes as well as from conservative statement to harmfulness rating by virtue of experimental randomization, but *not* for the paths from perceived harmfulness to the four outcomes which remain purely observational. We therefore control for individual political ideology which is related to both perceptions of harmfulness and willingness to cancel. In addition, we conducted a sensitivity analysis and found that it is unlikely that the ACMEs are explained by unobserved confounding (*SI Appendix*, Fig. S4). The results also hold when rebalancing the sample in terms of political ideology (*SI Appendix*, Tables S45–S47). We surveyed *N* = 1,233 German university students for Study 3. Further details on Study 3 and an additional Study 4 which manipulated a speaker’s ideological orientation without detailing the content of the speech are provided in *SI Appendix*.

## Supplementary Material

Appendix 01 (PDF)

## Data Availability

Replication data and code have been deposited in an OSF repository at https://osf.io/ezvm4/ (28).

## Footnotes

^*^We thank a reviewer for pointing out this important shortcoming in our original study.
