# Supplementary material for: Students’ motives for restricting academic freedom: Viewpoint discrimination and prosocial concerns
Source: Proc Natl Acad Sci U S A. 2025 Nov 20;122(47):e2503804122. doi: 10.1073/pnas.2503804122 (PMC12663967; doi:10.1073/pnas.2503804122)
Supplement: Supplementary file 1 — Appendix 01 (PDF) [file pnas.2503804122.sapp.pdf]

Supporting Information for
Students' motives for restricting academic freedom: Viewpoint discrimination and pro-social concerns

Claudia Diehl, Matthias Revers, Richard Traunmüller, Nils B. Weidmann, Alexander Wuttke

Richard Traunmüller
Email: traunmueller@uni-mannheim.de

This PDF file includes:

Supporting text
Tables S1 to S55
Figures S1 to S4
SI References

Details on the Adversarial Collaboration

We conducted an adversarial collaboration on the issue of free speech on university campuses with the participation of proponents (RT, MR) who concur with the basic conclusions in (*1*), and two groups of skeptics who do not agree. In addition, an impartial referee (AW), who takes a neutral stance, mediates between the groups in case of disagreement. This article tackles the critique of one team of opponents (CD, NW) and is the first in a series of papers to be published from this adversarial collaboration. The proponents and skeptics collaborated on a questionnaire and analysis plan for a quantitative survey that was conducted among university students in Germany. Before data collection, the proponents and skeptics committed bindingly to an adversarial collaboration agreement. The agreement documents the research design, data collection and analysis plan, along with the groups' hypotheses and the inference criteria under which hypotheses are supported or refuted.

We started with an initial evaluation of the original study (*1*) by conducting a brief survey among the scholars participating in the adversarial collaboration. They were asked to rate the conceptualization, sampling, measurement, and inference criteria of the research design using a 7-point scale ranging from 1 ('flawless') to 7 ('deficient'). Two main patterns emerged. As is to be expected, the critics gave the original research design worse ratings than did the original authors. Second, and more importantly, the ratings, including those of the authors of the original study, were predicted by social and political positioning. The more left-leaning the participant and the more intersecting minority characteristics, the more negatively the evaluation of the original research design. Considering that research is always influenced by the perspective of researchers, the current adversarial collaboration attempts to leverage the diversity of social positioning and political viewpoints between the groups to arrive at a more nuanced study than any of the individual researchers could produce by themselves.

Supporting Information for Study 1

Ethics. We obtained fully informed consent from all respondents. The study design was reviewed and approved by the ethics committee of the University of Mannheim (EK 28/2023).

Pre-registration. The study design and analysis plan were pre-registered prior to data collection: https://osf.io/mj942/?view_only=a5aca130ccbd4651bbbfcd0a5570c5e3

Deviations from the pre-analysis plan: We did not repeat the mini-surveys among participating researchers to rate the research design of the new study. In addition, to improve the flow of the argument, H1 and H2 are discussed in reverse order as compared to the pre-registration document.

Experimental Setup and Vignette Wording. We rely on a series of vignette experiments, which vary hypothetical scenarios along four dimensions: whether the statement is based on research or on the speaker's opinion, the content of the statement, whether the statement implies any policy recommendations, and whether there is mention of a minority group that criticizes it as offensive and discriminatory (see Table S1). Vignettes are constructed using a fully randomized 2^4 factorial design (note that content and policy recommendation depend on each other, thus yielding 4 distinct levels, rather than a true 2 x 2 randomization). We rely on a total of four vignette scenarios that address four different controversial topic areas: the role of biology in gender identity, women in STEM, the educational achievement of ethnic minority students, and the Muslim headscarf. Each respondent was asked to rate four fully randomized vignettes, referring to each of the four topic scenarios. The order in which these four topics were presented was fully randomized.

For each vignette, respondents are asked to judge four reactions of the university. They are asked whether a) the university should cancel the talk, b) the university should rescind a teaching position, c) the university should remove the speaker's book from the university library, and d) the university should allow protests. These outcomes were presented in a constant order. The answer categories are a simple binary yes/no response, respectively.

To avoid priming effects, the vignette experiments were asked at the very beginning of the questionnaire. They were only preceded by three neutral questions on students' study semester, their main subject as well as the federal state of their university. None of these questions are likely to influence the respondents' answers in the vignette experiments.

Table S1. Scenarios, attribute dimensions and levels of the vignettes in Study 1. The labels in parentheses indicate the respective variation of the statement. If a policy recommendation is included, it must correspond to the content of the speech, which is why there is a conservative and a progressive version of each policy recommendation. *The German word "Geschlecht" can be translated as either sex or gender.

Scenario	Gender identity	Women in STEM	Minority Student Achievement	Muslim Headscarf
Research vs. opinion	The journalist Mr. F. is invited to present his point of view. He argues *[opinion]*<br><br>Professor F. is invited to present the result of his research project. According to him, the research project found *[research]*			
Content of speech	that there are only two genders* and that belonging to one of them is biologically determined. *[conservative]*<br><br>that there are more than two genders and that belonging to one of them depends on one's identity. *[progressive]*	that the underrepresentation of women in technical subjects is primarily due to biologically determined preferences. *[conservative]*<br><br>that the underrepresentation of women in technical subjects is primarily due to sexist discrimination. *[progressive]*	that high school dropout rates among ethnic minorities are primarily due to their cultural characteristics. *[conservative]*<br><br>that high school dropout rates among ethnic minorities are primarily due to racial discrimination. *[progressive]*	that wearing an Islamic headscarf is usually not voluntary and is a sign of oppression. *[conservative]*<br><br>that wearing an Islamic headscarf is usually voluntary and can also be a sign of self-determination. *[progressive]*
Policy recommendation	He infers that access to women's restrooms should be reserved for those who were born biological women. *[conservative]*<br><br>He infers that each person should decide whether to use the men's or women's restroom based on his or her own gender	He infers that no anti-discrimination policy is necessary in this area. *[conservative]*<br><br>He infers that anti-discrimination policies are necessary in this area. *[progressive]*<br><br>*[No mention]*	He infers that anti-racism measures are not necessary. *[conservative]*<br><br>He infers that anti-racism measures are necessary. *[progressive]*<br><br>*[No mention]*	He concludes that it should be generally prohibited in public spaces. *[conservative]*<br><br>He concludes that it should generally be allowed in public spaces. *[progressive]*<br><br>*[No mention]*

<table>
<tr><td></td><td>identity.
[progressive]

[No mention]</td><td></td><td></td><td></td></tr>
<tr><td colspan="2">Reaction from university groups</td><td colspan="3">Some groups at the university criticize this statement as discriminatory and offensive.

[No mention]</td></tr>
</table>

Statistical Power. Statistical power analyses for multiple regression suggest that with the sample size of roughly N=1000 students and four vignettes per student (thus resulting in a stacked dataset of about N=4000 observations), assuming small effect sizes for the vignette characteristics of f^2 = .02 (*2*), and standard alpha levels of .05, the power to reject the null of no effects is no less than 1 (one). Conversely, the smallest detectable effect for our sample, assuming the standard power of .8, is around f^2 = .02 which corresponds to a tiny R^2 of .004. In short, we are almost certain to detect any effects of vignette characteristics if they indeed exist. Conversely, null findings indicate true null effects.

Data Collection and Sample. We commissioned the commercial survey company *forsa* with the data collection. The target population was German-speaking Internet users in Germany who are currently enrolled at a German university. The survey was conducted as part of *forsa.omninet*, a panel that is representative of the German online population aged 14 and over and currently has over 100,000 participants. The panel is recruited exclusively offline and based on a proper random probability sample which ensures equal and known inclusion probabilities. There is no possibility of self-selecting into the panel and participants' motivation to earn money is precluded. There are no multiple registrations, fake profiles, or automated bots. The panel also includes Internet non-users or partial offline users (e.g., people who only use e-mail, but no other online services).

The survey was fielded in the period from May 17 to July 17, 2023. To avoid selection bias, the purpose of the survey was introduced in a neutral way as 'studying university life and student attitudes in Germany'. Respondents were sent a maximum of three e-mail reminders. The participants received an incentive appropriate to the complexity and length of the survey. The current university student status was determined via a screening question. A gross sample of N=5420 was invited to participate out of which N=1144 (21%) were screened out because they were no longer students at a German university. Further N=3108 (57%) declined the invitation and N=165 (3%) dropped out before completing the questionnaire. The final sample of completed interviews consists of N=1003 respondents, resulting in a participation rate of 19% (or 23% among those who passed the initial screening question).

The sample for the vignette experiment was compared with official statistics of the German student population according to the German Federal Statistical Office (DESTATIS) as of March 10, 2023. Overall, the sample is a good representation of the current student population at German universities. In terms of subject or study program, students in STEM fields are slightly overrepresented (16% in the sample compared to 11% according to official student statistics) and engineering students underrepresented (17 compared to 26%). In addition, 9% report a residual unclassified study subject which in the population is close to zero. All remaining study fields, such as medicine, the fine arts, the humanities, or the social sciences, only deviate by a maximum of two percentage points from the population (see Table S2, Panel (A)). The sample covers a proportional number of students from all federal states in Germany. Only students from Baden-Württemberg are slightly overrepresented (16% vs. 12% in the population) and students from North Rhine-Westphalia are slightly underrepresented (22 vs. 26%. For details, see Table S2, Panel (B). The sample consists of 49% female students (51% in the population) with a median age of 25 years (23.5 in the population). First university semester students make up only 2% (compared to 7 in the population) and students with foreign citizenship only 1% (compared to 16% according to official statistics). Details are provided in Table S2, Panel (C).

Table S2. Representativeness of the sample. The sample for the vignette experiment was compared with official statistics of the German student population according to the German Federal Statistical Office (DESTATIS) as of March 10, 2023. This shows that the student sample is a good approximation of (A) the distribution of subjects, (B) the distribution of students across German states, and (C) the basic demographics in the German student population.

(A) Subject groups

	2021/2022		2022/2023*		Sample	
**Subject Group**	**N**	**Share**	**N**	**Share**	**N**	**Share**
Humanities	316442	0.11	312205	0.11	88	0.10
Sports	31157	0.01	30400	0.01	6	0.01
Law, Economics, and Social Sciences	1138785	0.39	1126170	0.39	337	0.37
Mathematics, Natural Sciences	314060	0.11	315473	0.11	143	0.16
Medicine/Health Sciences	196239	0.07	201258	0.07	58	0.06
Agrarian and Nutrition Sciences	64024	0.02	62927	0.02	18	0.02
Engineering	776744	0.26	765405	0.26	159	0.17
Arts, Fine Arts	100364	0.03	101483	0.03	25	0.03
Other Subjects	4100	0.00	8955	0.00	84	0.09
Total	2941915	1.00	2924276	1.00	918	1.00

(B) Federal states

	2021/2022		2022/2023*		Sample	
**Federal State**	**N**	**Share**	**N**	**Share**	**N**	**Share**
Baden-Württemberg	357342	0.12	354690	0.12	159	0.16
Bayern	404090	0.14	403437	0.14	157	0.16
Berlin	203869	0.07	198429	0.07	58	0.06
Brandenburg	50549	0.02	50443	0.02	21	0.02
Bremen	37393	0.01	37393	0.01	12	0.01
Hamburg	119110	0.04	119714	0.04	29	0.03
Hessen	262759	0.09	256216	0.09	79	0.08
Mecklenburg-Vorpommern	39041	0.01	38363	0.01	21	0.02
Niedersachsen	197246	0.07	197983	0.07	78	0.08
Nordrhein-Westfalen	764565	0.26	750501	0.26	218	0.22
Rheinland-Pfalz	121060	0.04	117009	0.04	43	0.04
Saarland	31835	0.01	30968	0.01	8	0.01
Sachsen	105868	0.04	106125	0.04	39	0.04
Sachsen-Anhalt	54823	0.02	58377	0.02	29	0.03
Schleswig-Holstein	67447	0.02	66150	0.02	19	0.02
Thüringen	124918	0.04	138478	0.05	22	0.02
Total	2941915	1.00	2924276	1.00	992	1.00

(C) Basic demographics

	2021/2022		2022/2023*		Sample	
**Demographics**	**N**	**Share**	**N**	**Share**	**N**	**Share**
First University Semester	214366	0.07	213428	0.07	24	0.02
Female	1475633	0.50	1476875	0.51	491	0.49
Foreign Citizenship	440564	0.15	458610	0.16	6	0.01
Median Age	23.5	--	n.a.	--	25	--

Balance Tests. We provide balance checks based on F-tests in Tables S3-S6. For each topic area we test for balance in the following covariates across all 16 experimental conditions: age, gender, foreign citizenship, first semester student, humanities, social sciences, STEM, southern federal state, eastern federal state, and city states (i.e. Berlin, Bremen, Hamburg). All experimental conditions are well balanced. The only exception is foreign citizenship in the minority student vignette. But this is due to the extremely small number of foreign students in the sample (N=6) and we therefore do not attempt to correct for this in our hypothesis tests.

Table S3. Balance test for gender identity vignette.

	Df	Sum Sq	Mean Sq	F value	Pr(> F)
Age	15	828.999	55.267	1.144	0.311
Female	15	4.950	0.330	1.325	0.179
Foreign Citizenship	15	0.059	0.004	0.658	0.827
First Semester	15	0.305	0.020	0.862	0.607
Humanities	15	1.062	0.071	0.813	0.663
Social Sciences	15	2.192	0.146	0.624	0.857
STEM	15	5.116	0.341	1.557	0.079
South	15	2.421	0.161	0.740	0.745
East	15	1.506	0.100	0.867	0.601
City States	15	1.720	0.115	1.281	0.207

Table S4. Balance test for women in STEM vignette.

	Df	Sum Sq	Mean Sq	F value	Pr(> F)
Age	15	714.256	47.617	0.984	0.470
Female	15	2.971	0.198	0.789	0.691
Foreign Citizenship	15	0.101	0.007	1.129	0.325
First Semester	15	0.155	0.010	0.436	0.969
Humanities	15	1.156	0.077	0.887	0.579
Social Sciences	15	2.176	0.145	0.620	0.860
STEM	15	3.510	0.234	1.060	0.390
South	15	2.559	0.171	0.782	0.698
East	15	1.642	0.109	0.947	0.511
City States	15	0.959	0.064	0.708	0.779

Table S5. Balance test for minority student achievement vignette.

	Df	Sum Sq	Mean Sq	F value	Pr(> F)
Age	15	791.043	52.736	1.091	0.360
Female	15	3.262	0.217	0.867	0.602
Foreign Citizenship	15	0.230	0.015	2.620	0.001
First Semester	15	0.293	0.020	0.828	0.647
Humanities	15	0.682	0.045	0.520	0.931
Social Sciences	15	2.757	0.184	0.788	0.693
STEM	15	1.234	0.082	0.368	0.986
South	15	1.639	0.109	0.499	0.942
East	15	0.693	0.046	0.397	0.980
City States	15	0.688	0.046	0.506	0.938

Table S6. Balance test for Muslim headscarf vignette.

	Df	Sum Sq	Mean Sq	F value	Pr(> F)
Age	15	968.214	64.548	1.341	0.171
Female	15	3.550	0.237	0.945	0.513
Foreign Citizenship	15	0.084	0.006	0.938	0.521
First Semester	15	0.459	0.031	1.304	0.192
Humanities	15	1.142	0.076	0.876	0.592
Social Sciences	15	4.755	0.317	1.371	0.154
STEM	15	2.528	0.169	0.760	0.724
South	15	2.766	0.184	0.847	0.625
East	15	1.137	0.076	0.653	0.832
City States	15	0.797	0.053	0.587	0.887

Attention/Manipulation Check. We also included an attention/manipulation check to evaluate how closely respondents read the vignette scenarios and whether they were aware of the key elements of the descriptions. To this end, we asked respondents to rate a fifth vignette like the previous four but related to the topic of anti-racism. Following this fifth vignette, respondents were asked four factual questions regarding this last vignette scenario: a) If you think about the last description, what was the profession of the invited speaker? [Professor; Journalist; Politician], b) If you think about the last description, does the invited speaker have any demands? [Yes, a political demand; Yes, another demand; No], c) If you think about the last description, what reaction did the invitation evoke? [A group criticized discrimination; The university administration demanded an apology; There was no reaction], and d) If you think about the last description, what was the general political thrust of the speaker's statement? [Progressive; Conservative; No political thrust]. Correct answers to each of these questions were given by 65%, 76%, 68%, and 56%, respectively. This indicates that clear majorities of our respondents had read and understood the vignette descriptions and were therefore experimentally manipulated as intended. Based on the answers to these four test questions, we constructed an attentiveness index by summing up the false responses. The resulting index runs from 0 to 4, where higher values indicate less attentiveness. Detecting either high levels of inattentiveness (60% of our respondents made a maximum of one mistake (see Table S7)) or any sample differences between attentive and non-attentive respondents (there are none, see Table S8) would serve informational purposes only and would not lead to any corrections in our hypothesis tests. Importantly, to avoid post-treatment bias, we do not exclude respondents who did not pass the attention checks in our main analysis (*3*).

Table S7. Number of wrong answers in attention check.

Number of Wrong Answers	0	1	2	3	4
Share	0.22	0.38	0.26	0.13	0.01
N	211	372	254	125	12

Table S8. Testing determinants of attention in the survey. The table shows OLS regression results with the attentiveness index (0-4) as the dependent variable.

Age	-0.001
	(0.005)
Female	0.075
	(0.068)
Foreign citizenship	0.419
	(0.449)
First university semester	0.144
	(0.216)
Humanities	-0.031
	(0.131)
Social Sciences	0.090
	(0.092)
STEM	0.163
	(0.095)
South	0.016
	(0.078)
East	-0.023
	(0.104)
City State	0.014
	(0.119)
Constant	1.234
	(0.167)
N	893
R^2	0.008

Results.

Table S9. Main regression results of vignette experiments. The table shows results of linear probability models with the four vignette outcomes. Standard errors are clustered at the respondent level. Topic FEs are effect-coded to allow for an interpretation of the intercept as overall mean across topics. *p < .05; **p < .01

	Model 1: Cancel Talk at University	Model 2: Rescind Teaching Position	Model 3: Remove Book from Library	Model 4: Allow Disruptive Protest
Intercept	-0.016<br>(0.013)	-0.011<br>(0.011)	-0.009<br>(0.009)	0.452**<br>(0.021)
Opinion	0.065**<br>(0.013)	0.056**<br>(0.011)	0.036**<br>(0.010)	0.025<br>(0.016)
Conservative statement	0.312**<br>(0.015)	0.253**<br>(0.013)	0.168**<br>(0.012)	0.206**<br>(0.017)
Policy recommendation	0.072**<br>(0.013)	0.047**<br>(0.011)	0.040**<br>(0.011)	0.041**<br>(0.015)
Reaction from groups on campus	0.015<br>(0.012)	-0.021+<br>(0.011)	0.004<br>(0.010)	0.040*<br>(0.015)
Topic FEs	yes	yes	yes	yes
R^2	0.16	0.13	0.08	0.05
N	3963	3906	3824	3902

Table S10. Testing the ideological balance of the vignette statements. The table shows regression results (linear probability models) where respondents' assessment of whether a statement is true is predicted by their ideological self-placement (11-point scale; 0: left, 10: right). This measure was standardized such that an increase by one unit corresponds to a shift in political ideology by one standard deviation. Ideological balance holds if for conservative statements (top panel) we find significant positive coefficients and if for the progressive statements (bottom panel), we find significant negative coefficients, and if the magnitude of these coefficients is similar. The signs of the coefficients have the expected directions, but perfect balance applies only in the case of the first and the last vignette topic. $^{*}p < .05$; $^{**}p < .01$

	Two Biological Genders	Women in STEM: Biology	Minority Students: Culture	Muslim Headscarf: Oppression
Left-Right-Ideology	0.21^{**} (0.01)	0.12^{**} (0.01)	0.12^{**} (0.01)	0.05^{**} (0.02)
Intercept	0.28^{**} (0.01)	0.20^{**} (0.01)	0.17^{**} (0.01)	0.33^{**} (0.02)
R^2	0.22	0.09	0.11	0.01
N	933	932	934	902
	More Than Two Genders	Women in STEM: Discrimination	Minority Students: Discrimination	Muslim Headscarf: Self-determination
Left-Right-Ideology	-0.22^{**} (0.01)	-0.19^{**} (0.01)	-0.20^{**} (0.01)	-0.05^{**} (0.02)
Intercept	0.73^{**} (0.01)	0.45^{**} (0.02)	0.40^{**} (0.01)	0.52^{**} (0.02)
R^2	0.25	0.15	0.16	0.01
N	937	929	921	899

Table S11. Main results using ideologically balanced statements only. Regression results from a linear probability model. $^{*}p < .05$; $^{**}p < .01$

	Model 1: Cancel Talk at University	Model 2: Rescind Teaching Position	Model 3: Remove Book from Library	Model 4: Allow Disruptive Protest
Intercept	0.018<br>(0.019)	0.024<br>(0.017)	0.027<br>(0.015)	0.470**<br>(0.028)
Opinion	0.041*<br>(0.017)	0.054**<br>(0.015)	0.028*<br>(0.014)	0.007<br>(0.022)
Conservative statement	0.312**<br>(0.018)	0.225**<br>(0.017)	0.157**<br>(0.015)	0.181**<br>(0.023)
Policy recommendation	0.081**<br>(0.017)	0.047**<br>(0.015)	0.021<br>(0.014)	0.048*<br>(0.021)
Reaction from groups on campus	0.005<br>(0.017)	-0.023<br>(0.015)	-0.000<br>(0.014)	0.052*<br>(0.021)
Left-Right-Ideology	-0.044**<br>(0.009)	-0.030**<br>(0.008)	-0.018*<br>(0.008)	-0.073**<br>(0.014)
Topic FEs	yes	yes	yes	yes
R^2	0.17	0.12	0.09	0.06
N	1957	1946	1904	1941

Table S12. Main results of vignette experiments with survey weights included. The table shows regression results (linear probability models) with standard errors clustered by respondents. Topic FEs are effect-coded to allow for an interpretation of the intercept as overall mean across topics. $^{*}p < .05$; $^{**}p < .01$

	Model 1: Cancel Talk at University	Model 2: Rescind Teaching Position	Model 3: Remove Book from Library	Model 4: Allow Disruptive Protest
Intercept	-0.016<br>(0.013)	-0.011<br>(0.011)	-0.009<br>(0.009)	0.452**<br>(0.021)
Opinion	0.065**<br>(0.013)	0.056**<br>(0.011)	0.036**<br>(0.010)	0.025<br>(0.016)
Conservative statement	0.312**<br>(0.015)	0.253**<br>(0.013)	0.168**<br>(0.012)	0.206**<br>(0.017)
Policy recommendation	0.072**<br>(0.013)	0.047**<br>(0.011)	0.040**<br>(0.011)	0.041**<br>(0.015)
Reaction from groups on campus	0.015<br>(0.012)	-0.021+<br>(0.011)	0.004<br>(0.010)	0.040*<br>(0.015)
Topic FEs	yes	yes	yes	yes
R^2	.16	.13	.08	.05
N	3963	3906	3824	3902

Table S13. Main results of vignette experiments adjusting for attentiveness. The table shows regression results (linear probability models) with standard errors clustered by respondents. Topic FEs are effect-coded to allow for an interpretation of the intercept as overall mean across topics. $^{*}p < .05$; $^{**}p < .01$

	Model 1: Cancel Talk at University	Model 2: Rescind Teaching Position	Model 3: Remove Book from Library	Model 4: Allow Disruptive Protest
Intercept	-0.015<br>(0.016)	-0.013<br>(0.014)	-0.016<br>(0.013)	0.474**<br>(0.027)
Opinion	0.066**<br>(0.013)	0.058**<br>(0.011)	0.036**<br>(0.010)	0.026+<br>(0.016)
Conservative statement	0.312**<br>(0.015)	0.255**<br>(0.014)	0.167**<br>(0.012)	0.205**<br>(0.017)
Policy recommendation	0.071**<br>(0.013)	0.045**<br>(0.011)	0.039**<br>(0.011)	0.038*<br>(0.015)
Reaction from groups on campus	0.017<br>(0.012)	-0.020+<br>(0.011)	0.004<br>(0.010)	0.040**<br>(0.016)
Attentiveness Index	-0.001<br>(0.008)	0.001<br>(0.007)	0.006<br>(0.007)	-0.015<br>(0.013)
Topic FEs	yes	yes	yes	yes
R^2	.16	.14	.08	.05
N	3870	3842	3762	3840

Table S14. Analysis of vignette experiment with Logit models. The table shows regression results (Logit models) with standard errors clustered by respondents. Topic FEs are effect-coded to allow for an interpretation of the intercept as overall mean across topics. *p < .05; **p < .01

	Model 1: Cancel Talk at University	Model 2: Rescind Teaching Position	Model 3: Remove Book from Library	Model 4: Allow Disruptive Protest
Intercept	-3.333** (0.153)	-3.878** (0.188)	-3.924** (0.193)	-0.213** (0.088)
Opinion	0.458** (0.089)	0.493** (0.097)	0.386** (0.108)	0.112 (0.069)
Conservative statement	2.252** (0.126)	2.552** (0.170)	2.061** (0.171)	0.887** (0.076)
Policy recommendation	0.511** (0.090)	0.417** (0.097)	0.432** (0.114)	0.180** (0.067)
Reaction from groups on campus	0.104 (0.085)	-0.187* (0.095)	0.039 (0.107)	0.177* (0.068)
Topic FEs	yes	yes	yes	yes
N	3963	3906	3824	3902

Table S15. Main results of vignette experiments controlling for ideological self-placement (all vignette topics). The table shows regression results (linear probability models) with standard errors clustered by respondents. Topic FEs are effect-coded to allow for an interpretation of the intercept as overall mean across topics. *p < .05; **p < .01

	Model 1: Cancel Talk at University	Model 2: Rescind Teaching Position	Model 3: Remove Book from Library	Model 4: Allow Disruptive Protest
Intercept	-0.013<br>(0.013)	-0.008<br>(0.011)	-0.008<br>(0.009)	0.459**<br>(0.021)
Opinion	0.063**<br>(0.013)	0.055**<br>(0.011)	0.035**<br>(0.010)	0.022<br>(0.015)
Conservative statement	0.312**<br>(0.015)	0.255**<br>(0.013)	0.169**<br>(0.012)	0.208**<br>(0.017)
Policy recommendation	0.071**<br>(0.013)	0.045**<br>(0.011)	0.039**<br>(0.010)	0.035*<br>(0.015)
Reaction from groups on campus	0.012<br>(0.012)	-0.024*<br>(0.011)	0.002<br>(0.010)	0.037*<br>(0.015)
Left-Right-Ideology	-0.043**<br>(0.007)	-0.040**<br>(0.007)	-0.021**<br>(0.007)	-0.075**<br>(0.013)
Topic FEs	yes	yes	yes	yes
R^2	0.17	0.15	0.08	0.07
N	3911	3881	3800	3877

Table S16. Main regression results of vignette experiments re-weighted to match a 50:50 distribution between left-leaning and right-leaning students. The table shows results of linear probability models with the four vignette outcomes. Standard errors are clustered at the respondent level. Topic FEs are effect-coded to allow for an interpretation of the intercept as overall mean across topics. $^{*}p < .05$; $^{**}p < .01$

	Model 1: Cancel Talk at University	Model 2: Rescind Teaching Position	Model 3: Remove Book from Library	Model 4: Allow Disruptive Protest
Intercept	-0.002	0.003	-0.003	0.445***
	(0.014)	(0.011)	(0.011)	(0.024)
Opinion	0.061**	0.048**	0.038**	0.031*
	(0.014)	(0.011)	(0.011)	(0.018)
Conservative statement	0.251**	0.202**	0.132**	0.156**
	(0.016)	(0.014)	(0.013)	(0.019)
Policy recommendation	0.077**	0.048**	0.047**	0.049**
	(0.014)	(0.012)	(0.011)	(0.017)
Reaction from groups on campus	0.018	-0.018	0.006	0.030*
	(0.013)	(0.011)	(0.011)	(0.018)
Topic FEs	yes	yes	yes	yes
R^2	0.11	0.10	0.06	0.03
N	3911	3881	3800	3877

Table S17. Main results of vignette experiments controlling for subject of study (social science vs. others). The table shows regression results (linear probability models) with standard errors clustered by respondents. Topic FEs are effect-coded to allow for an interpretation of the intercept as overall mean across topics. *p < .05; **p < .01

	Model 1: Cancel Talk at University	Model 2: Rescind Teaching Position	Model 3: Remove Book from Library	Model 4: Allow Disruptive Protest
Intercept	-0.037*<br>(0.015)	-0.029*<br>(0.013)	-0.026*<br>(0.011)	0.452**<br>(0.025)
Opinion	0.066**<br>(0.013)	0.058**<br>(0.012)	0.037**<br>(0.011)	0.021<br>(0.016)
Conservative statement	0.314**<br>(0.015)	0.258**<br>(0.014)	0.167**<br>(0.013)	0.203**<br>(0.018)
Policy recommendation	0.072**<br>(0.013)	0.052**<br>(0.012)	0.043**<br>(0.011)	0.048**<br>(0.016)
Reaction from groups on campus	0.016<br>(0.013)	-0.021+<br>(0.011)	0.002<br>(0.010)	0.036*<br>(0.016)
Social Sciences	0.057**<br>(0.016)	0.041**<br>(0.015)	0.047**<br>(0.013)	0.002<br>(0.027)
Topic FEs	yes	yes	yes	yes
R^2	0.17	0.12	0.09	0.06
N	1957	1946	1904	1941

Table S18. Main results of vignette experiments controlling for science telos (understanding vs. changing the world). The table shows regression results (linear probability models) with standard errors clustered by respondents. Topic FEs are effect-coded to allow for an interpretation of the intercept as overall mean across topics. $^{*}p < .05$; $^{**}p < .01$

	Model 1: Cancel Talk at University	Model 2: Rescind Teaching Position	Model 3: Remove Book from Library	Model 4: Allow Disruptive Protest
Intercept	0.025<br>(0.021)	0.045*<br>(0.020)	0.009<br>(0.018)	0.496**<br>(0.035)
Opinion	0.065**<br>(0.013)	0.056**<br>(0.011)	0.036**<br>(0.010)	0.025<br>(0.016)
Conservative statement	0.312**<br>(0.015)	0.254**<br>(0.013)	0.168**<br>(0.012)	0.207**<br>(0.017)
Policy recommendation	0.072**<br>(0.013)	0.045**<br>(0.011)	0.039**<br>(0.010)	0.040**<br>(0.015)
Reaction from groups on campus	0.015<br>(0.012)	-0.021*<br>(0.011)	0.004<br>(0.010)	0.041**<br>(0.015)
Science: understand the world	-0.049*<br>(0.020)	-0.066**<br>(0.020)	-0.021<br>(0.019)	-0.052<br>(0.034)
Topic FEs	yes	yes	yes	yes
R^2	0.17	0.12	0.09	0.06
N	1957	1946	1904	1941

Table S19. Vignette Interactions: Conservative statement x Opinion vs. Research. The table shows regression results (linear probability models) with standard errors clustered by respondents. Topic FEs are effect-coded to allow for an interpretation of the intercept as overall mean across topics. *p < .05; **p < .01

	Model 1: Cancel Talk at University	Model 2: Rescind Teaching Position	Model 3: Remove Book from Library	Model 4: Allow Disruptive Protest
Intercept	0.010<br>(0.012)	0.014<br>(0.009)	0.0001<br>(0.008)	0.457**<br>(0.023)
Opinion	0.014<br>(0.011)	0.008<br>(0.007)	0.018*<br>(0.007)	0.016<br>(0.022)
Conservative statement	0.260**<br>(0.018)	0.204**<br>(0.015)	0.149**<br>(0.014)	0.196**<br>(0.022)
Policy recommendation	0.072**<br>(0.013)	0.047**<br>(0.011)	0.040**<br>(0.011)	0.041**<br>(0.015)
Reaction from groups on campus	0.015<br>(0.012)	-0.021<br>(0.011)	0.004<br>(0.010)	0.040**<br>(0.015)
Conservative statement x Opinion	0.102**<br>(0.025)	0.096**<br>(0.022)	0.036<br>(0.019)	0.019<br>(0.029)
Topic FEs	yes	yes	yes	yes
N	3936	3906	3824	3902

Table S20. Vignette Interactions: Conservative statement x policy recommendation. The table shows regression results (linear probability models) with standard errors clustered by respondents. Topic FEs are effect-coded to allow for an interpretation of the intercept as overall mean across topics. $^{*}p < .05$; $^{**}p < .01$

	Model 1: Cancel Talk at University	Model 2: Rescind Teaching Position	Model 3: Remove Book from Library	Model 4: Allow Disruptive Protest
Intercept	0.021*<br>(0.012)	0.011<br>(0.010)	0.009<br>(0.009)	0.452***<br>(0.023)
Opinion	0.065**<br>(0.013)	0.056**<br>(0.011)	0.036**<br>(0.010)	0.025<br>(0.016)
Conservative statement	0.237**<br>(0.018)	0.210**<br>(0.016)	0.131**<br>(0.015)	0.207**<br>(0.023)
Policy recommendation	-0.001<br>(0.011)	0.004<br>(0.008)	0.004<br>(0.008)	0.041<br>(0.022)
Reaction from groups on campus	0.015<br>(0.012)	-0.021<br>(0.011)	0.004<br>(0.010)	0.040**<br>(0.015)
Conservative statement x Policy recommendation	0.146**<br>(0.024)	0.084**<br>(0.021)	0.072**<br>(0.020)	-0.001<br>(0.031)
Topic FEs	yes	yes	yes	yes
N	3936	3906	3824	3902

Table S21. Vignette Interactions: Conservative statement x Reaction from groups on campus. The table shows regression results (linear probability models) with standard errors clustered by respondents. Topic FEs are effect-coded to allow for an interpretation of the intercept as overall mean across topics. $^{*}p < .05$; $^{**}p < .01$

	Model 1: Cancel Talk at University	Model 2: Rescind Teaching Position	Model 3: Remove Book from Library	Model 4: Allow Disruptive Protest
Intercept	-0.009 (0.012)	-0.015 (0.010)	-0.002 (0.009)	0.433** (0.023)
Opinion	0.065** (0.013)	0.056** (0.011)	0.036** (0.010)	0.025 (0.016)
Conservative statement	0.297** (0.019)	0.263** (0.018)	0.153** (0.016)	0.245** (0.023)
Policy recommendation	0.072** (0.013)	0.047** (0.011)	0.040** (0.011)	0.041** (0.015)
Reaction from groups on campus	0.001 (0.011)	-0.012 (0.008)	-0.011 (0.008)	0.078** (0.023)
Conservative statement x Reaction from groups on campus	0.028 (0.025)	-0.019 (0.021)	0.030 (0.020)	-0.076* (0.029)
Topic FEs	yes	yes	yes	yes
N	3936	3906	3824	3902

Table S22. Full Vignette Interactions. The table shows regression results (linear probability models) with standard errors clustered by respondents. Topic FEs are effect-coded to allow for an interpretation of the intercept as overall mean across topics. $^{*}p < .05$; $^{**}p < .01$

	Model 1: Cancel Talk at University	Model 2: Rescind Teaching Position	Model 3: Remove Book from Library	Model 4: Allow Disruptive Protest
Intercept	0.062** (0.015)	0.042** (0.013)	0.026* (0.010)	0.446** (0.033)
Opinion	-0.010 (0.020)	-0.022 (0.016)	-0.002 (0.014)	0.032 (0.045)
Conservative statement	0.166** (0.032)	0.149** (0.029)	0.087** (0.023)	0.208** (0.044)
Policy recommendation	-0.005 (0.021)	-0.017 (0.017)	0.005 (0.016)	0.050 (0.047)
Reaction from groups on campus	-0.004 (0.022)	-0.018 (0.016)	-0.003 (0.014)	0.070 (0.045)
Opinion x Policy recommendation	0.022 (0.031)	0.055* (0.025)	0.033 (0.025)	-0.078 (0.064)
Opinion x Reaction from groups on campus	0.025 (0.031)	0.029 (0.020)	0.022 (0.021)	-0.048 (0.064)
Policy recommendation x Reaction from groups on campus	-0.014 (0.031)	0.009 (0.021)	-0.020 (0.020)	-0.032 (0.066)
Opinion x Conservative statement	0.133** (0.047)	0.119** (0.043)	0.064 (0.037)	0.005 (0.061)
Policy recommendation x Conservative statement	0.162** (0.046)	0.131** (0.042)	0.088* (0.037)	0.038 (0.062)
Reaction from groups on campus x Conservative statement	0.006 (0.045)	-0.004 (0.039)	0.026 (0.035)	-0.058 (0.061)
Opinion x Policy x Reaction	0.001 (0.046)	-0.046 (0.032)	-0.031 (0.032)	0.185* (0.091)
Opinion x Policy x Conservative	-0.069 (0.069)	-0.047 (0.063)	-0.043 (0.058)	0.055 (0.085)
Opinion x Reaction x Conservative	0.007 (0.069)	0.015 (0.060)	-0.003 (0.055)	0.101 (0.089)
Policy x Reaction x Conservative	0.036 (0.066)	-0.034 (0.058)	0.016 (0.053)	-0.007 (0.088)
Opinion x Policy x Reaction x Conservative	-0.006 (0.097)	-0.027 (0.086)	-0.015 (0.082)	-0.248* (0.124)
Topic FEs	yes	yes	yes	yes
N	3936	3906	3824	3902

Supporting Information for Study 2

Ethics. We obtained fully informed consent from all respondents. The study design was reviewed and approved by the ethics committee of the University of Mannheim (EK 16/2025).
Pre-registration. The study design and analysis plan were pre-registered prior to data collection, see https://osf.io/dxce9.
Pretest. For a prior assessment of the ideological position and the perceived harmfulness of the statements, we conducted a pretest on a different sample, together with Study 4. The results presented in Tables S27 and S28 and Figure S1 are based on this sample, which is described in detail in the supporting information for Study 4 below.
Experimental Setup and Vignette Wording. The revised set of vignettes for Study 2 uses the two most balanced vignettes from Study 1 and adds a new vignette topic (Israel vs. Palestine). The vignettes now include detailed information about the potential harm that the proposed university talks could entail. The wording of the vignettes is presented in Table S23. The vignette outcomes are the same as in Study 1.

Table S23. Scenarios, attribute dimensions and levels of the vignettes in Study 2. The labels in parentheses indicate the respective variation of the statement. Note that unlike in Study 1, the vignettes include a specific description of the potential harm that the statements could entail.

Scenario	Gender Identity	Muslim Headscarf	Israel vs. Palestine
Opinion vs. research	The journalist Mr. F. is invited to present his point of view. He argues *[opinion]*<br><br>Professor F. is invited to present the result of his research project. According to him, the research project found *[research]*		
Conservative vs. progressive statement without/with harm statement	that there are only two genders and that belonging to one of the two genders is biologically determined. *[conservative, without harm statement]*<br><br>that there are only two genders and that belonging to one of the two genders is biologically determined. Some groups at the university criticize this statement as harmful, because it can lead people to live in the wrong body, which can later cause psychological crises. *[conservative, with harm statement]*<br><br>that there are more than two genders and that belonging to one gender depends on one's identity.	that wearing an Islamic headscarf is a sign of oppression and is usually not done voluntarily. *[conservative, without harm statement]*<br><br>that wearing an Islamic headscarf is a sign of oppression and is usually not done voluntarily. Some groups at the university criticize this statement as harmful, because it puts pressure on Muslim women to avoid wearing a headscarf even though they would prefer that. *[conservative, with harm statement]*<br><br>that wearing an Islamic headscarf is a sign of self-determination and usually done voluntarily.	that what the Palestinians are doing to the Israelis is terrorism. *[conservative, without harm statement]*<br><br>that what the Palestinians are doing to the Israelis is terrorism. Some groups at the university criticize this statement as harmful, because it fosters islamophobia, which can lead to violence against Muslims. [*conservative, with harm statement]*<br><br>that what the Israelis are doing to the Palestinians is

	[progressive, without harm statement]<br><br>that there are more than two genders and that belonging to one gender depends on one's identity. Some groups at the university criticize this statement as harmful, because it can lead people to change their body through hormones or operations, which can later cause psychological crises. [progressive, with harm statement]	[progressive, without harm statement]<br><br>that wearing an Islamic headscarf is a sign of self-determination and usually done voluntarily. Some groups at the university criticize this statement as harmful, because it puts pressure on Muslim women to wear a headscarf against their will. [progressive, with harm statement]	apartheid. [progressive, without harm statement]<br><br>that what the Israelis are doing to the Palestinians is apartheid. Some groups at the university criticize this statement as harmful, because it fosters antisemitism, which can lead to violence against Jews. [progressive, with harm statement]

Statistical Power. Statistical power analyses for multiple regression suggest that with the sample size of roughly N=1000 students and three vignettes per student (thus resulting in a stacked data set of about N=3000 observations), assuming small effect sizes for the vignette characteristics of $f^2 = .02$ (*2*), and standard alpha levels of .05, the power to reject the null of no effects is .999. Conversely, the smallest detectable effect for our sample, assuming the standard power of .8, is around $f^2 = .004$ which corresponds to a tiny R^2 of .004. In short, we have near-certain power to detect any effects of vignette characteristics if they indeed exist. Conversely, null findings indicate true null effects.

Data Collection and Sample. Data were collected via Prolific from a sample of 1,111 German university students. The survey was conducted between November 15, 2024, and December 9, 2024. The median age is 24 years of age, 46.5 percent of respondents are female, 13.7 percent first semester students, and 34.7 have a foreign citizenship.

Balance Tests. We provide balance checks based on F-tests in Tables S24-S26. For each topic area we test for balance in the following covariates: age, gender, foreign citizenship, first semester student and left-right ideological self-placement. All experimental conditions are well balanced, except for gender and foreign citizenship in the Israel-Palestine vignette, where we find some imbalances across treatments. However, we refrain from correcting these by controlling for respondents' gender and citizenship in the analyses.

Table S24. Balance tests for gender identity vignette in Study 2.

	Df	Sum Sq	Mean Sq	F value	Pr(> F)
Age	7	105.953	15.136	0.679	0.690
Female	7	1.376	0.197	0.789	0.597
Foreign Citizenship	7	2.217	0.317	1.404	0.200
First Semester	7	0.603	0.086	0.730	0.647
Left-Right-Ideology	7	35.535	5.076	1.413	0.196

Table S25. Balance test for Muslim headscarf vignette in Study 2.

	Df	Sum Sq	Mean Sq	F value	Pr(> F)
Age	7	317.139	45.306	2.050	0.046
Female	7	1.999	0.286	1.148	0.330
Foreign Citizenship	7	1.063	0.152	0.670	0.697
First Semester	7	0.351	0.050	0.425	0.887
Left-Right-Ideology	7	12.775	1.825	0.505	0.831

Table S26. Balance test for Israel/Palestine vignette in Study 2.

	Df	Sum Sq	Mean Sq	F value	Pr(> F)
Age	7	106.086	15.155	0.680	0.689
Female	7	3.836	0.548	2.218	0.031
Foreign Citizenship	7	4.035	0.576	2.574	0.012
First Semester	7	0.462	0.066	0.559	0.790
Left-Right-Ideology	7	24.631	3.519	0.977	0.447

Table S27. Testing the ideological position of the vignette statements in Study 2. The table shows respondents' average placement of vignette statements on the ideological left-right scale (10-point scale; 1: left, 10: right).

	Two Biological Genders	Muslim Headscarf: Oppression	Israel/Palestine: Terrorism
Left-Right-Ideology	7.99	6.69	6.37
N	623	625	624
	More Than Two Genders	Muslim Headscarf: Self-determination	Israel/Palestine: Apartheid
Left-Right-Ideology	2.68	4.01	4.34
N	627	624	623

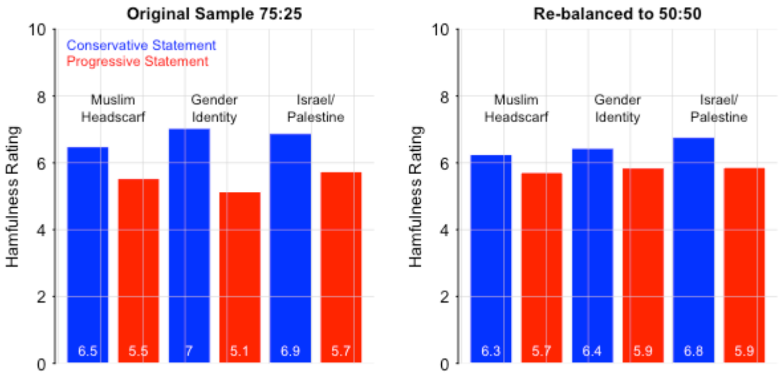

Figure S1. Respondents' assessment of the harmfulness of the vignette statements used in Study 2 (11-point scale; 0: not at all harmful, 10: extremely harmful). The left panel shows the average harmfulness ratings. Perceived harm is slightly higher for conservative than for progressive statements (between 1.0 and 1.9 points on scale from 0-10). However, harm ratings are not objective but subjectively biased by the ideological preferences of raters, whose distribution in the sample is clearly skewed in favor of left compared to the right (75:25 ratio). The right panel shows the average harm ratings in a re-balanced sample with a 50:50 ratio. The gap in the perceived harm of conservative and progressive reduces to around half a scale point.

Table S28. Testing whether the potential harm implied by conservative and progressive statements is similar. The table shows regression results (OLS) where respondents' assessment of how harmful a statement is (11-point scale; 0: not at all harmful, 10: extremely harmful) is predicted by their ideological self-placement. Similar levels of harm hold if for conservative statements (top panel) we find significant negative coefficients and if for the progressive statements (bottom panel), we find significant positive coefficients, and if the magnitude of these coefficients is similar. The signs of the coefficients have the expected directions, equal levels of perceived harm apply only in the case of the first and the last vignette topic. $^{*}p < .05$; $^{**}p < .01$

	Two Biological Genders	Muslim Headscarf: Oppression	Israel/Palestine: Terrorism
Left-Right-Ideology	-2.74**	-1.26**	-1.10**
	(0.23)	(0.21)	(0.22)
Intercept	7.04**	6.49**	6.88**
	(0.12)	(0.11)	(0.11)
R^2	0.18	0.05	0.04
N	623	625	624
	More Than Two Genders	Muslim Headscarf: Self-determination	Israel/Palestine: Apartheid
Left-Right-Ideology	2.97**	0.64**	0.81**
	(0.23)	(0.21)	(0.22)
Intercept	5.12**	5.53**	5.74**
	(0.12)	(0.10)	(0.11)
R^2	0.21	0.02	0.02
N	627	624	623

Attention/Manipulation Check. To check whether the vignette dimensions indeed manipulated the desired constructs, we included an attention/manipulation check after the final vignette. Respondents were asked a) what the political stance of the last statement was and b) whether it was considered harmful by minority groups. The results presented in Table S29 demonstrate that vignettes with a conservative statement were significantly and substantively perceived as conservative, whereas the social harm manipulation did not impact the perceived political stance. More importantly and regarding the perceived social harm of the vignette scenarios, we note three things. First, even if no explicit manipulation of harm was included, 45% believed the statement mentioned harm to minority groups. Second, the manipulated conservative versus progressive vignette statements do not differ significantly whether respondents considered them harmful. Third, the explicit manipulation of social harm significantly increased respondents' perception of harm by 40 percentage points. Taken together this suggests that respondents indeed impute harm into statements when it is not made explicit, but that this is not specific to conservative statements and therefore does not explain their effects and finally that we were successful in manipulating harm perceptions.

Table S29. Results of attention/manipulation check. The table shows regression results (OLS) with standard errors of two experimental manipulations on remembered 'political stance' and 'social harm' of the last vignette. $^{*}p < .05$; $^{**}p < .01$

	Conservative	Harmful
Manipulation: Conservative Statement	0.373^{**}	0.029
	(0.027)	(0.026)
Manipulation: Social Harm	0.025	0.402^{**}
	(0.027)	(0.026)
Constant	0.214^{**}	0.446^{**}
	(0.024)	(0.022)
N	1112	1113
R^2	0.144	0.182

Results.

Table S30. Main regression results of the vignette experiments. The table shows results of linear probability models with the four vignette outcomes. Standard errors are clustered at the respondent level. Topic FEs are effect-coded to allow for an interpretation of the intercept as overall mean across topics. *p < .05; **p < .01

	Model 1: Cancel Talk at University	Model 2: Rescind Teaching Position	Model 3: Remove Book from Library	Model 4: Allow Disruptive Protest
Intercept	0.099** (0.013)	0.050** (0.011)	0.075** (0.011)	0.605** (0.019)
Opinion	0.028 (0.014)	0.044** (0.012)	0.018 (0.012)	0.031* (0.015)
Conservative statement	0.198** (0.015)	0.141** (0.013)	0.100** (0.012)	0.101** (0.016)
Social harm	0.042** (0.014)	0.018 (0.012)	0.004 (0.012)	0.051** (0.016)
Topic FEs	yes	yes	yes	yes
R^2	0.06	0.04	0.02	0.02
N	3432	3435	3426	3439

Table S31. Vignette interactions: Conservative statement x social harm. The table shows regression results (linear probability models) with standard errors clustered by respondents. Topic FEs are effect-coded to allow for an interpretation of the intercept as overall mean across topics. $^{*}p < .05$; $^{**}p < .01$

	Model 1: Cancel Talk at University	Model 2: Rescind Teaching Position	Model 3: Remove Book from Library	Model 4: Allow Disruptive Protest
Intercept	0.070**	0.092**	0.048**	0.609**
	(0.012)	(0.013)	(0.011)	(0.020)
Opinion	0.018	0.028	0.044**	0.031*
	(0.012)	(0.014)	(0.012)	(0.015)
Conservative statement	0.110**	0.211**	0.144**	0.094**
	(0.016)	(0.020)	(0.018)	(0.023)
Social harm	0.015	0.055**	0.021*	0.044*
	(0.013)	(0.016)	(0.013)	(0.023)
Conservative statement x Social harm	-0.021	-0.026	-0.006	0.015
	(0.023)	(0.027)	(0.024)	(0.031)
Topic FEs	yes	yes	yes	yes
R^2	0.07	0.04	0.02	0.02
N	3432	3435	3426	3439

Table S32. Main results of vignette experiment controlling for ideological self-placement (all vignette topics). The table shows regression results (linear probability models) with standard errors clustered by respondents. Topic FEs are effect-coded to allow for an interpretation of the intercept as overall mean across topics. $^{*}p < .05$; $^{**}p < .01$

	Model 1: Cancel Talk at University	Model 2: Rescind Teaching Position	Model 3: Remove Book from Library	Model 4: Allow Disruptive Protest
Intercept	0.190**	0.127**	0.097**	0.729**
	(0.021)	(0.019)	(0.019)	(0.030)
Opinion	0.026	0.042**	0.017	0.029
	(0.014)	(0.012)	(0.012)	(0.015)
Conservative statement	0.199**	0.141**	0.100**	0.102**
	(0.015)	(0.013)	(0.012)	(0.016)
Social harm	0.040**	0.018	0.004	0.050**
	(0.014)	(0.012)	(0.012)	(0.015)
Respondent left-right ideology	-0.021**	-0.018**	-0.005	-0.029**
	(0.004)	(0.004)	(0.004)	(0.006)
Topic FEs	yes	yes	yes	yes
R^2	0.07	0.05	0.02	0.03
N	3429	3432	3424	3436

Table S33. Main results of study 2 vignette experiments re-weighted to match a 50:50 distribution between left-leaning and right-leaning students. The table shows regression results (linear probability models) with standard errors clustered by respondents. Topic FEs are effect-coded to allow for an interpretation of the intercept as overall mean across topics. $^{*}p < .05$; $^{**}p < .01$

	Model 1: Cancel Talk at University	Model 2: Rescind Teaching Position	Model 3: Remove Book from Library	Model 4: Allow Disruptive Protest
Constant	0.590**	0.119**	0.064**	0.093**
	(0.023)	(0.016)	(0.013)	(0.014)
Opinion	0.037	0.021	0.041**	0.012
	(0.020)	(0.017)	(0.014)	(0.015)
Conservative statement	0.050*	0.137**	0.096**	0.066**
	(0.020)	(0.017)	(0.014)	(0.014)
Social harm	0.063**	0.036*	0.019	0.009
	(0.019)	(0.016)	(0.013)	(0.014)
Topic FEs	yes	yes	yes	yes
R^2	0.04	0.02	0.01	0.01
N	3432	3435	3426	3439

Table S34. Vignette Interactions: Conservative statement x opinion x social harm. The table shows regression results (linear probability models) with standard errors clustered by respondents. Topic FEs are effect-coded to allow for an interpretation of the intercept as overall mean across topics. *p < .05; **p < .01

	Model 1: Cancel Talk at University	Model 2: Rescind Teaching Position	Model 3: Remove Book from Library	Model 4: Allow Disruptive Protest
Intercept	0.097**	0.066**	0.073**	0.632**
	(0.015)	(0.013)	(0.013)	(0.025)
Opinion	0.017	0.009	0.012	-0.013
	(0.021)	(0.017)	(0.019)	(0.033)
Conservative statement	0.186**	0.111**	0.086**	0.057*
	(0.027)	(0.023)	(0.022)	(0.033)
Social harm	0.066**	0.023	0.027	0.006
	(0.022)	(0.018)	(0.019)	(0.034)
Opinion x Conservative	0.049	0.064*	0.049	0.071
	(0.038)	(0.032)	(0.032)	(0.045)
Opinion x Harm	-0.021	-0.004	-0.023	0.074
	(0.031)	(0.025)	(0.026)	(0.047)
Conservative x Harm	-0.019	-0.016	-0.009	0.074
	(0.038)	(0.031)	(0.032)	(0.045)
Opinion x Conservative x Harm	-0.014	0.020	-0.025	-0.116
	(0.054)	(0.047)	(0.045)	(0.062)
Topic FEs	yes	yes	yes	yes
R^2	0.07	0.05	0.02	0.02
N	3432	3435	3426	3439

Supporting Information for Study 3

Ethics. We obtained fully informed consent from all respondents. The study design was reviewed and approved by the ethics committee of the University of Mannheim (Supplement 1 to EK 16/2025).

Pre-registration. The study design and analysis plan were pre-registered prior to data collection, see https://osf.io/d6sz9.

Experimental Setup and Vignette Wording. The experimental setup and vignette wordings in study 3 are an exact replication of study 2 (see Table S23). In addition, after each experimental vignette we asked respondents to rate the presented statements using the following two questions: a) *On a scale of 0 (not harmful at all) to 10 (very harmful), how harmful do you consider the content of the planned lecture to be?* b) *On a scale from 0 (far left) to 10 (far right), where would you place the content of the planned lecture politically?*

Statistical Power. Statistical power analyses for multiple regression suggest that with the sample size of roughly N=1000 students and three vignettes per student (thus resulting in a stacked data set of about N=3000 observations), assuming small effect sizes for the vignette characteristics of $f^2 = .02$ (*2*), and standard alpha levels of .05, the power to reject the null of no effects is .999. Conversely, the smallest detectable effect for our sample, assuming the standard power of .8, is around $f^2 = .004$ which corresponds to a tiny R^2 of .004. In short, we are almost certain to detect any effects of vignette characteristics if they indeed exist. Conversely, null findings indicate true null effects.

Data Collection and Sample. We commissioned the survey company *Bilendi* to provide a sample of German university students. The survey was conducted between August 13th and August 21st 2025 and yielded a final sample of N=1233 respondents currently enrolled in a German university. The median age is 23 years of age, 53.8 percent of respondents are female, 16.1 percent first semester students, and 6.8 have a foreign citizenship.

Balance Tests. We provide balance checks based on F-tests in Tables S35-S37. For each topic area we test for balance in the following covariates: age, gender, foreign citizenship, first semester student and left-right ideological self-placement. All experimental conditions are well balanced.

Table S35. Balance tests for gender identity vignette in Study 3.

	Df	Sum Sq	Mean Sq	F value	Pr(> F)
Age	7	239.348	34.193	0.994	0.434
Female	7	1.831	0.262	1.052	0.393
Foreign Citizenship	7	0.227	0.032	0.508	0.829
First Semester	7	0.714	0.102	0.755	0.625
Left-Right-Ideology	7	37.805	5.401	1.165	0.320

Table S36. Balance test for Muslim headscarf vignette in Study 3.

	Df	Sum Sq	Mean Sq	F value	Pr(> F)
Age	7	328.332	46.905	1.367	0.216
Female	7	0.888	0.127	0.509	0.828
Foreign Citizenship	7	0.562	0.080	1.265	0.264
First Semester	7	1.303	0.186	1.383	0.209
Left-Right-Ideology	7	26.049	3.721	0.801	0.586

Table S37. Balance test for Israel/Palestine vignette in Study 3.

	Df	Sum Sq	Mean Sq	F value	Pr(> F)
Age	7	271.827	38.832	1.130	0.341
Female	7	2.318	0.331	1.334	0.231
Foreign Citizenship	7	0.224	0.032	0.503	0.833
First Semester	7	0.988	0.141	1.047	0.396
Left-Right-Ideology	7	36.107	5.158	1.112	0.353

Manipulation Check. After each experimental vignette we asked respondents to rate the presented statements according to their perceived harmfulness and their perceived political position. The model specification in study 3 is close to the one in study 2 with two crucial differences: a) the dependent variables are *respondents' own ratings*, and b) these ratings are asked after *each of the three presented vignettes*. If ideology and harm can be separated, we would observe that the 'conservative statement' manipulation has a strong and significant effect on the 'political position' rating but no effect on the 'harmfulness' rating. Conversely, we should observe that the 'social harm' manipulation has a strong and significant effect on the 'harmfulness' rating but no effect on the 'political position' rating. The results of manipulation check presented in Table S38 confirms that the experimental conditions largely affect the intended constructs, but also reveals notable spillovers between them. In short, respondents appear to associate conservative views with harm and vice versa.

Table S38. Results of manipulation check for Study 3. The table shows regression results (OLS) with clustered standard errors of experimental manipulations on individual respondents' 'harmfulness' and 'political position' ratings. $^{*}p < .05$; $^{**}p < .01$

	Harmfulness rating	Political position rating
Manipulation: Opinion vs. Research	-0.005	-0.070
	(0.088)	(0.092)
Manipulation: Conservative Statement	1.098^{**}	1.724^{**}
	(0.095)	(0.092)
Manipulation: Social Harm	0.504^{**}	0.235^{**}
	(0.089)	(0.085)
Constant	4.351^{**}	3.996^{**}
	(0.092)	(0.096)
Topic FEs	yes	yes
N	3429	3075
R^2	0.078	0.116

Results.

Table S39. Main regression results of the vignette experiments in Study 3. The table shows results of linear probability models with the four vignette outcomes. Standard errors are clustered at the respondent level. Topic FEs are effect-coded to allow for an interpretation of the intercept as overall mean across topics. $^{*}p < .05$; $^{**}p < .01$

	Model 1: Cancel Talk at University	Model 2: Rescind Teaching Position	Model 3: Remove Book from Library	Model 4: Allow Disruptive Protest
Intercept	0.229**	0.193**	0.214**	0.559**
	(0.016)	(0.015)	(0.015)	(0.018)
Opinion	0.029	0.040**	0.011	-0.006
	(0.015)	(0.015)	(0.015)	(0.016)
Conservative statement	0.127**	0.091**	0.103**	0.076**
	(0.016)	(0.015)	(0.015)	(0.017)
Social harm	0.049**	0.032*	0.042**	0.062**
	(0.016)	(0.015)	(0.015)	(0.016)
Topic FEs	yes	yes	yes	yes
R^2	0.025	0.016	0.017	0.014
N	3676	3667	3640	3678

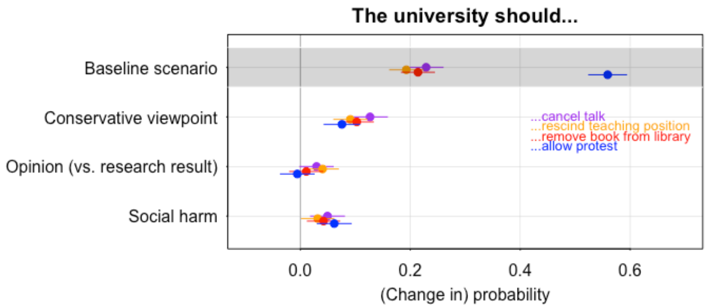

Fig. S2. Main results of the vignette experiment in Study 3. Estimated effects of journalist opinion (vs. professor research), conservative (vs. progressive) viewpoint, and the presence of potential social harm associated with the speaker's viewpoint. Coefficients and 95% confidence intervals are from a linear probability model with clustered standard errors and vignette topic fixed effects. The baseline scenario (first line) corresponds to progressive research results without an explicit mention of harm. Detailed results are provided in Table S39.

Table S40. Main regression results of the vignette experiments in Study 3 re-weighted to match a 50:50 distribution between left-leaning and right-leaning students. The table shows results of linear probability models with the four vignette outcomes. Standard errors are clustered at the respondent level. Topic FEs are effect-coded to allow for an interpretation of the intercept as overall mean across topics. $^{*}p < .05$; $^{**}p < .01$

	Model 1: Cancel Talk at University	Model 2: Rescind Teaching Position	Model 3: Remove Book from Library	Model 4: Allow Disruptive Protest
Intercept	0.266**	0.220**	0.234**	0.560**
	(0.020)	(0.020)	(0.019)	(0.022)
Opinion	0.037*	0.051**	0.017	-0.013
	(0.018)	(0.018)	(0.018)	(0.019)
Conservative statement	0.056**	0.038*	0.063**	0.051**
	(0.019)	(0.019)	(0.019)	(0.020)
Social harm	0.036	0.024	0.049**	0.060**
	(0.019)	(0.018)	(0.018)	(0.019)
Topic FEs	yes	yes	yes	yes
R^2	0.025	0.016	0.017	0.014
N	3676	3667	3640	3678

Table S41. Vignette Interactions: Conservative statement x opinion x social harm. The table shows regression results (linear probability models) with standard errors clustered by respondents. Topic FEs are effect-coded to allow for an interpretation of the intercept as overall mean across topics. *p < .05; **p < .01

	Model 1: Cancel Talk at University	Model 2: Rescind Teaching Position	Model 3: Remove Book from Library	Model 4: Allow Disruptive Protest
Intercept	0.233***	0.213***	0.225***	0.568***
	(0.020)	(0.021)	(0.021)	(0.024)
Opinion	0.028	0.002	-0.008	-0.017
	(0.028)	(0.028)	(0.028)	(0.032)
Conservative statement	0.085***	0.043	0.072**	0.063**
	(0.030)	(0.028)	(0.029)	(0.032)
Social harm	0.046	0.004	0.025	0.045
	(0.029)	(0.028)	(0.028)	(0.031)
Opinion x Conservative	0.073*	0.092**	0.052	0.010
	(0.043)	(0.041)	(0.040)	(0.046)
Opinion x Harm	-0.006	0.050	0.026	0.018
	(0.042)	(0.040)	(0.041)	(0.045)
Conservative x Harm	0.075*	0.069*	0.049	0.020
	(0.043)	(0.040)	(0.042)	(0.045)
Opinion x Conservative x Harm	-0.125**	-0.129**	-0.081	-0.011
	(0.063)	(0.059)	(0.061)	(0.063)
Topic FEs	yes	yes	yes	yes
R^2	0.027	0.017	0.017	0.014
N	3676	3667	3640	3678

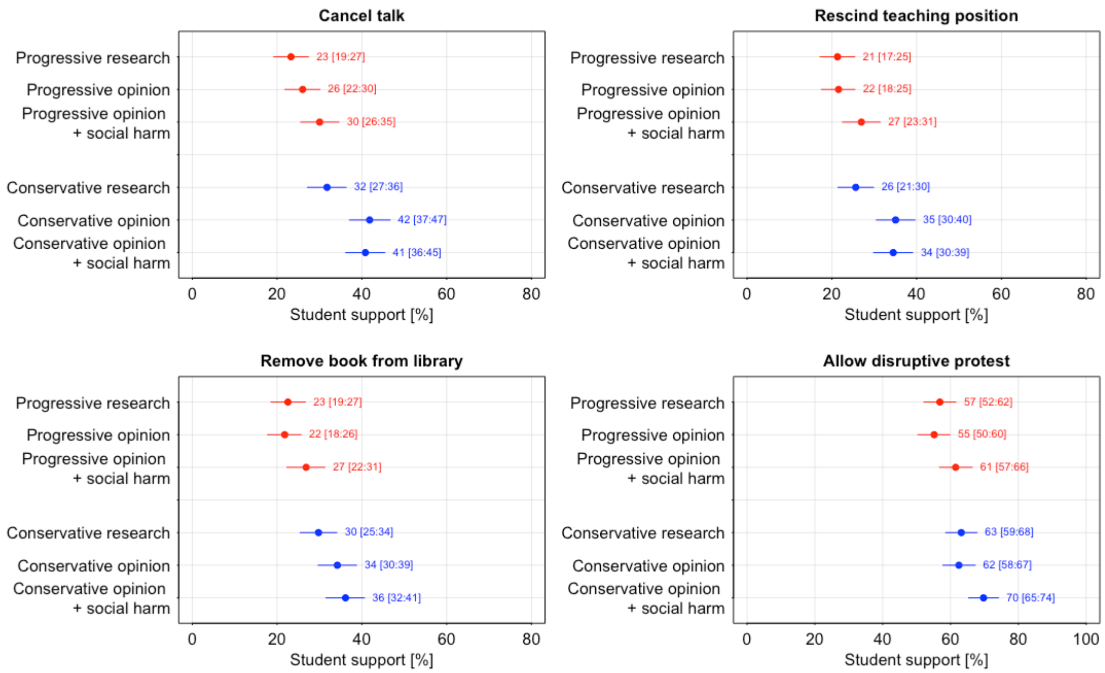

Fig. S3. Predicted probabilities for support of canceling talks, rescinding teaching positions, removing books, and allowing protests in Study 3. Marginal effects and simulated 95% confidence intervals are based on a linear probability model with clustered standard errors and vignette-topic fixed effects. Detailed results are provided in Table S41.

Table S42. Causal mediation analysis. Outcome model equations controlling for respondent ideology. The table shows results of linear probability models with the four vignette outcomes. Standard errors are clustered at the respondent level. Topic FEs are effect-coded to allow for an interpretation of the intercept as overall mean across topics. $^{*}p < .05$; $^{**}p < .01$

	Model 1: Cancel Talk at University	Model 2: Rescind Teaching Position	Model 3: Remove Book from Library	Model 4: Allow Disruptive Protest
Intercept	-0.030	-0.022	-0.039	0.503^{**}
	(0.026)	(0.028)	(0.026)	(0.032)
Opinion	0.032^{*}	0.044^{**}	0.011	-0.009
	(0.015)	(0.015)	(0.016)	(0.016)
Conservative statement	0.047^{**}	0.029	0.038^{*}	0.038^{*}
	(0.016)	(0.016)	(0.016)	(0.017)
Social harm	0.004	0.0003	0.007	0.049^{**}
	(0.016)	(0.015)	(0.015)	(0.016)
Harmfulness rating	0.069^{**}	0.052^{**}	0.057^{**}	0.033^{**}
	(0.003)	(0.004)	(0.003)	(0.004)
Respondent ideology	-0.003	0.003	0.008	-0.017^{**}
	(0.004)	(0.005)	(0.005)	(0.005)
Topic FEs	yes	yes	yes	yes
R^2	0.157	0.101	0.115	0.049
N	3343	3332	3311	3343

Table S43. Causal mediation analysis: Mediation model equations. The table shows regression results (OLS) with clustered standard errors of experimental manipulations on individual respondents' 'harmfulness' ratings. $^{*}p < .05$; $^{**}p < .01$

	Model 1: Harmfulness rating	Model 2: Harmfulness rating	Model 3: Harmfulness rating	Model 4: Harmfulness rating
Manipulation: Opinion vs. Research	-0.002	-0.007	-0.018	-0.003
	(0.088)	(0.088)	(0.088)	(0.087)
Manipulation: Conservative Statement	1.098**	1.090**	1.113**	1.108**
	(0.088)	(0.088)	(0.088)	(0.088)
Manipulation: Social Harm	0.511**	0.504**	0.507**	0.505**
	(0.087)	(0.088)	(0.088)	(0.087)
Constant	4.353**	4.365**	4.363**	4.347**
	(0.088)	(0.088)	(0.088)	(0.088)
Topic FEs	yes	yes	yes	yes
N	3343	3332	3311	3343
R^2	0.080	0.079	0.080	0.080

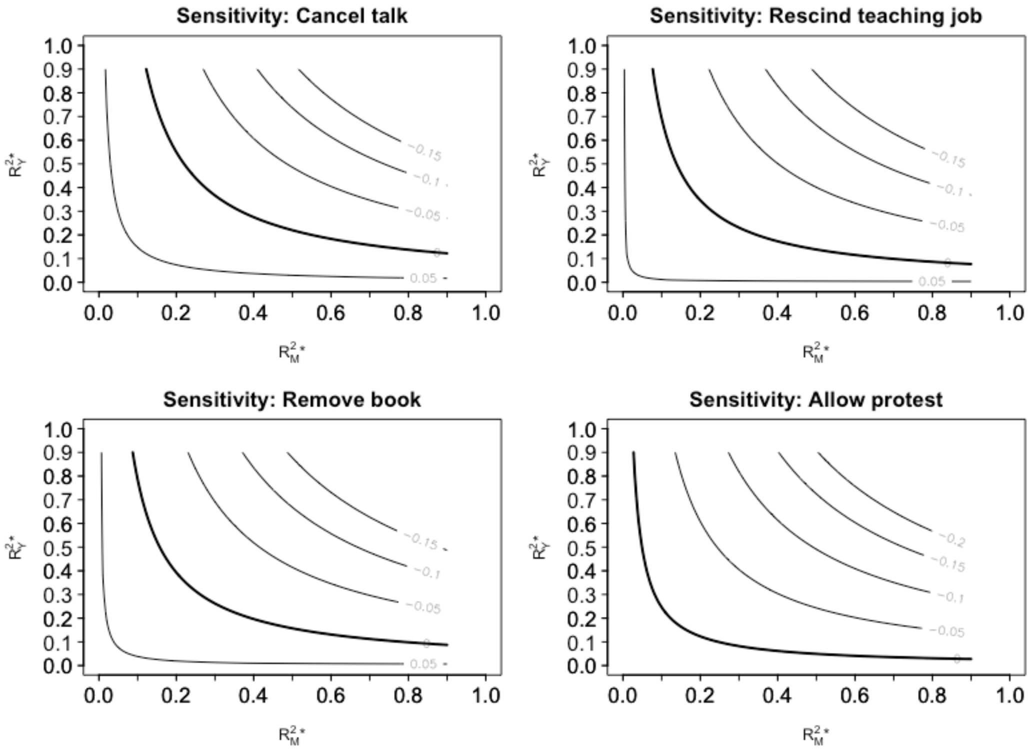

Figure S4. Sensitivity Analysis for causal mediation analysis. ACMEs for hypothetical values for the residual R^2s of an unobserved confounder explaining the mediator (perceived harmfulness rating) and the four outcomes. This sensitivity contour plot helps to explore the robustness of an estimate to a confounder at different strengths. The horizontal axis shows the partial R2 of the unobserved confounder with the treatment. The vertical axis shows the partial R2 of the unobserved confounder with the outcome. The contour levels show the adjusted estimate one would have obtained in the full regression adjusting for the unobserved confounder with such strength. The bold line is a critical threshold: confounders with such strength (or stronger) are sufficient to invalidate the research conclusions.

Table S44. Causal mediation analysis: Quantities of interest. Derived from the outcome and mediation equations in tables S42 and S43. Quasi-Bayesian 95% credible intervals based on S=10000 simulations.

	Cancel Talk at University	Rescind Teaching Position	Remove Book from Library	Allow Disruptive Protest
ACME	0.076	0.057	0.063	0.036
	[0.063:0.090]	[0.046:0.068]	[0.052:0.075]	[0.028:0.046]
ADE	0.047	0.029	0.038	0.038
	[0.017:0.078]	[-0.001:0.059]	[0.008:0.069]	[0.004:0.071]
Total effect	0.123	0.086	0.102	0.074
	[0.091:0.156]	[0.055:0.117]	[0.070:0.133]	[0.041:0.107]
Proportion mediated	0.619	0.661	0.621	0.488
	[0.484:0.823]	[0.476:1.018]	[0.464:0.892]	[0.319:0.913]

Table S45. Causal mediation analysis: Outcome model equations controlling for respondent ideology re-weighted to match a 50:50 distribution between left-leaning and right-leaning students. The table shows results of linear probability models with the four vignette outcomes. Standard errors are clustered at the respondent level. Topic FEs are effect-coded to allow for an interpretation of the intercept as overall mean across topics. $^{*}p < .05$; $^{**}p < .01$

	Model 1: Cancel Talk at University	Model 2: Rescind Teaching Position	Model 3: Remove Book from Library	Model 4: Allow Disruptive Protest
Intercept	-0.034	-0.047	-0.063*	0.493**
	(0.033)	(0.034)	(0.032)	(0.035)
Opinion	0.044*	0.058**	0.020	-0.013
	(0.018)	(0.018)	(0.019)	(0.020)
Conservative statement	0.020	0.012	0.039*	0.042*
	(0.019)	(0.019)	(0.019)	(0.020)
Social harm	-0.006	-0.004	0.019	0.051**
	(0.019)	(0.018)	(0.018)	(0.019)
Harmfulness rating	0.070**	0.054**	0.055**	0.032**
	(0.004)	(0.004)	(0.004)	(0.004)
Respondent ideology	-0.001	0.005	0.011*	-0.014**
	(0.005)	(0.005)	(0.005)	(0.005)
Topic FEs	yes	yes	yes	yes
R^2	0.149	0.103	0.107	0.042
N	3343	3332	3311	3343

Table S46. Causal mediation analysis: Mediation model equations re-weighted to match a 50:50 distribution between left-leaning and right-leaning students. The table shows regression results (OLS) with clustered standard errors of experimental manipulations on individual respondents' 'harmfulness' ratings. $^{*}p < .05$; $^{**}p < .01$

	Model 1: Harmfulness rating	Model 2: Harmfulness rating	Model 3: Harmfulness rating	Model 4: Harmfulness rating
Manipulation: Opinion vs. Research	-0.050	-0.053	-0.064	-0.051
	(0.088)	(0.089)	(0.089)	(0.088)
Manipulation: Conservative Statement	0.603**	0.599**	0.607**	0.619**
	(0.088)	(0.089)	(0.089)	(0.088)
Manipulation: Social Harm	0.455**	0.451**	0.463**	0.453**
	(0.088)	(0.089)	(0.089)	(0.088)
Constant	4.741**	4.751**	4.748**	4.732**
	(0.088)	(0.088)	(0.088)	(0.088)
Topic FEs	yes	yes	yes	yes
N	3343	3332	3311	3343
R^2	0.048	0.048	0.048	0.048

Table S47. Causal mediation analysis: Quantities of interest re-weighted to match a 50:50 distribution between left-leaning and right-leaning students. Derived from the outcome and mediation equations in Tables S45 and S46. Quasi-Bayesian 95% credible intervals based on S=10000 simulations.

	Cancel Talk at University	Rescind Teaching Position	Remove Book from Library	Allow Disruptive Protest
ACME	0.020	0.042	0.033	0.034
	[0.014:0.027]	[0.030:0.054]	[0.023:0.043]	[0.024:0.044]
ADE	0.043	0.019	0.011	0.039
	[0.010:0.074]	[-0.010:0.047]	[-0.018:0.043]	[0.009:0.069]
Total effect	0.063	0.060	0.044	0.072
	[0.029:0.096]	[0.029:0.091]	[0.011:0.075]	[0.042:0.104]
Proportion mediated	0.315	0.686	0.744	0.459
	[0.189:0.697]	[0.450:1.374]	[0.411:2.400]	[0.309:0.812]

Table S48. Results of Study 3 including an interaction between conservative statement and respondents' harmfulness ratings. The table shows results of linear probability models with the four vignette outcomes. Standard errors are clustered at the respondent level. Topic FEs are effect-coded to allow for an interpretation of the intercept as overall mean across topics. $^{*}p < .05$; $^{**}p < .01$

	Model 1: Cancel Talk at University	Model 2: Rescind Teaching Position	Model 3: Remove Book from Library	Model 4: Allow Disruptive Protest
Intercept	-0.018	-0.026	-0.001	0.455**
	(0.022)	(0.022)	(0.022)	(0.030)
Opinion	0.031*	0.045**	0.012	-0.010
	(0.015)	(0.015)	(0.016)	(0.016)
Conservative statement	-0.002	0.061	0.023	-0.010
	(0.030)	(0.033)	(0.031)	(0.038)
Social harm	0.004	-0.0002	0.007	0.049**
	(0.016)	(0.015)	(0.015)	(0.016)
Harmfulness rating	0.064**	0.056**	0.056**	0.027**
	(0.004)	(0.005)	(0.004)	(0.005)
Conservative statement x Harmfulness rating	0.010	-0.006	0.003	0.010
	(0.006)	(0.006)	(0.006)	(0.006)
Topic FEs	yes	yes	yes	yes
R^2	0.157	0.101	0.113	0.044
N	3349	3338	3316	3349

Study 4: Canceling Ideology or Content?

Studies 1 to 3 converge in the conclusion that viewpoint discrimination is the primary motivation for students' willingness to restrict academic freedom. In Study 4, we ask whether viewpoint discrimination persists even when no information is provided about the content of a talk, and only the ideological orientation of the event or speaker is manipulated. Rather than teasing out and balancing harm as in studies 2 and 3, Study 4 takes the opposite approach by studying cancel culture based solely on political labels, not content. If viewpoint discrimination operates similarly to what was observed in the previous studies, we should find that events or individuals associated with conservative ideology are more likely to be targeted for cancelation. To test this, we present abstract hypothetical scenarios that are devoid of substantive content and include only information about the speaker's/author's ideological affiliation. Details on the study design and the wording of the vignettes are provided in the SI (Table S49-S55).

Ethics. We obtained fully informed consent from all respondents. The study design was reviewed and approved by the ethics committee of the University of Mannheim (EK 16/2025).

Pre-registration. The study design and analysis plan were pre-registered prior to data collection, see https://osf.io/537yz/?view_only=ca23595a997045e89c2292463fe5fed9.

Experimental Setup and Vignette Wording. We present German university students with three hypothetical scenarios: (1) a speaking event organized by a political student group, (2) a discussion about offering a teaching position to a controversial scientist, and (3) the potential removal of a book from the university library. All three experiments followed a simple 1 x 2 between-subjects design, randomizing whether the guest speaker, scientist, or author was described as politically progressive or conservative. Further details on power analyses, data collection, sampling, and balance checks are provided below.

The table below shows the different vignettes used in Study 4. The experimental treatment is *[displayed in square brackets].*

Table S49 Scenarios, attribute dimensions and levels of the vignettes in Study 4. The labels in parentheses indicate the respective variation of the statement.

Cancel event scenario	*Revoke teaching position scenario*	*Remove book scenario*
You now see a short description of a fictitious incident at your university. Please read this description and tell us what you think the university should do.<br><br>The *[conservative Association of Christian Democratic Students // left-wing Socialist Student Union]* invites a guest speaker on a politically highly controversial topic. The guest speaker is known for his particularly *[conservative // left-wing]* viewpoints. Some groups at the university fear that unacceptable statements will be made at the event and	You will now see another description of a fictitious incident at your university. Please read this description and tell us what you think the university should do.<br><br>There is a controversial discussion at your university about the awarding of a teaching position to a *[conservative // left-wing]* academic who has recently attracted attention in the public eye due to his prominent statements. He has repeatedly expressed *clearly [conservative // left-wing]* positions that some groups at the	You will now see a final description of a fictitious incident at your university. Please read this description and tell us what you think the university should do.<br><br>Some groups at the university are calling for the removal of a book by a *[conservative // left-wing]* author on a highly controversial political topic that was recently added to the university library. The book takes a clearly *[conservative // leftist]* stance. Some groups argue that there is unacceptable content in the book.

are therefore calling for the event to be canceled.	university find unacceptable.	
Should the university cancel the event?<br><br>Response: Yes/No	Should the university revoke the teaching position already awarded to this person?<br><br>Response: Yes/No	Should the book be removed from the university library?<br><br>Response: Yes/No

Statistical Power. A power analysis using a two-sample t-test with N=300 per experimental group suggests that the minimally detectable effect with a conventional power of .8 and an alpha level of .05 is a Cohen's d of about .229 which is just above what is considered a 'small' effect size (*2*). This corresponds to a percentage point difference of about 11 percentage points.
Data Collection and Sample. Data were collected via Prolific from a sample of 620 German university students. The survey was conducted between September 27, 2024, and October 5, 2024.
Balance tests. We provide balance checks based on F-tests in Tables S45-S47. For each topic area we test for balance in age, gender, and left-right ideological self-placement. All experimental conditions are well balanced.

Table S50. Balance test for the cancel talk vignette in Study 3.

	Df	Sum Sq	Mean Sq	F value	Pr(> F)
Age	1	12.772	12.772	0.470	0.493
Female	1	0.067	0.067	0.269	0.604
Left-Right-Ideology	1	1.246	1.246	0.361	0.548

Table S51. Balance test for the teaching position vignette in Study 3.

	Df	Sum Sq	Mean Sq	F value	Pr(> F)
Age	1	12.107	12.107	0.446	0.505
Female	1	0.058	0.058	0.231	0.631
Left-Right-Ideology	1	10.698	10.698	3.113	0.078

Table S52. Balance test for the remove book vignette in Study 3.

	Df	Sum Sq	Mean Sq	F value	Pr(> F)
Age	1	48.228	48.228	1.779	0.183
Female	1	0.235	0.235	0.939	0.333
Left-Right-Ideology	1	1.823	1.823	0.528	0.468

Results. Across all three scenarios, the experimental evidence points to viewpoint discrimination against conservative speakers or events (see Table S53 for results). Even in the absence of specific content, students are significantly more likely to support the cancelation of an event organized by a conservative rather than a socialist student group, to support rescinding a teaching position from a conservative rather than a progressive scientist, and to support the removal of a book by a conservative rather than a left-wing author. We again assess whether these results could be explained by the ideological imbalance in our sample, where approximately three quarters of respondents (76%) place themselves at 5 or below on a 1–10 left–right self-placement scale. Controlling for political ideology does not alter the results (Table S54). When reweighting the sample to simulate a 50:50 distribution of left- and right-leaning students, support for denying a teaching position to a controversial scientist remains substantially higher when the scientist is conservative, though differences for the other two outcomes become less pronounced (Table S55).

Table S53. Main results of the vignette experiment in Study 3. The table shows regression coefficients (OLS) with standard errors; $^{*}p < .05$; $^{**}p < .01$

	Model 1: Cancel Talk at University	Model 2: Rescind Teaching Position	Model 3: Remove Book from Library
Treatment: Left-leaning	-0.136**	-0.239**	-0.080**
	(0.035)	(0.037)	(0.026)
Constant	0.340**	0.459**	0.165**
	(0.026)	(0.026)	(0.019)
R^2	0.023	0.064	0.015
N	617	616	616

Table S54. Main results of vignette experiment in Study 3, controlling for ideological self-placement (all vignette topics). The table shows regression results (linear probability models) with standard errors clustered by respondents. Topic FEs are effect-coded to allow for an interpretation of the intercept as overall mean across topics. $^{*}p < .05$; $^{**}p < .01$

	Model 1: Cancel Talk at University	Model 2: Rescind Teaching Position	Model 3: Remove Book from Library
Treatment: Left-leaning	-0.135**	-0.245**	-0.080**
	(0.035)	(0.037)	(0.027)
Respondent: Left-right ideology	-0.011	-0.022*	0.0003
	(0.010)	(0.010)	(0.007)
Constant	0.385**	0.556**	0.164**
	(0.047)	(0.051)	(0.036)
R^2	0.025	0.071	0.015
N	617	616	616

Table S55. Main results of the 'pure ideology experiments' re-weighted to match a 50:50 distribution between left-leaning and right-leaning students. The table shows regression coefficients (OLS) with standard errors; $^{*}p < .05$; $^{**}p < .01$

	Model 1: Cancel Talk at University	Model 2: Rescind Teaching Position	Model 3: Remove Book from Library
Treatment: Left-leaning	-0.034	-0.166^{***}	-0.037
	(0.036)	(0.037)	(0.027)
Constant	0.282^{***}	0.388^{***}	0.143^{***}
	(0.026)	(0.025)	(0.019)
R^2	0.001	0.032	0.003
N	617	616	616

SI References

1. M. Revers, R. Traunmüller, Is Free Speech in Danger on University Campus? Some Preliminary Evidence from a Most Likely Case. *Köln Z Soziol* **72**, 471–497 (2020).

2. J. Cohen, *Statistical power analysis for the behavioral sciences* (Lawrence Erlbaum, Hillsdale, NS, 2nd edition, 1988).

3. J. M. Montgomery, B. Nyhan, M. Torres, How Conditioning on Posttreatment Variables Can Ruin Your Experiment and What to Do about It. *American J Political Sci* **62**, 760–775 (2018).
